# Zinc Speciation in Fine and Coarse Fly Ash Particles Collected In-Flight at a Waste Incinerator

**DOI:** 10.1021/acsenvironau.5c00222

**Published:** 2026-01-30

**Authors:** Evelina Gorjatšova, Fanny Bergman, Kajsa G. V. Sigfridsson Clauss, Nils Skoglund, Karin Karlfeldt Fedje, Jenny Rissler

**Affiliations:** † Ergonomics and Aerosol Technology, Department of Design Sciences, 5193 Lund University, Lund SE-22100, Sweden; ‡ MAX IV Laboratory, Lund University, Box 118, Lund SE-22100, Sweden; § Thermochemical Energy Conversion Laboratory, Department of Applied Physics and Electronics, 98833 Umeå University, Umeå SE-901 87, Sweden; ∥ Recycling and Waste Management, Renova AB, Box 156, Gothenburg SE-401 22, Sweden; ⊥ Department of Architecture and Civil Engineering, Chalmers University of Technology, Gothenburg SE-412 96, Sweden; # NanoLund, Lund University, Lund SE-22100, Sweden

**Keywords:** municipal solid waste incineration, zinc speciation, aerosol, XANES, size separation, waste-to-energy

## Abstract

Safe and optimized utilization of waste-to-energy (WtE) fly ash (FA) requires a detailed understanding of the physicochemical properties of its metal constituents. This study provides a comprehensive analysis of the chemical form of Zn in fine (<1 μm) and coarse (>1 μm) FA particles, hypothesized to originate from different formation mechanisms. Size-selective aerosol sampling was performed during standard operation in the flue gas channel at a WtE facility. Additionally, FA samples from the air pollution control filters at the facility and boiler deposits were analyzed. Speciation was determined primarily using synchrotron-based X-ray absorption spectroscopy, complemented by XRD, SEM-EDS, and total elemental analysis. Significant differences in terms of elemental composition, crystalline phases, and Zn chemical forms were observed between fine- and coarse FA particles. Fine particles were dominated by Cl, K, and Na with Zn almost exclusively present as potassium zinc chlorides. Coarse particles were heterogeneous, with Zn occurring in stable forms such as aluminate, ferrite, and silicates (e.g., gehlenite). The major elemental constituents were Ca, Si, and Al. Although coarse particles constitute the major mass of the FA, about 50% of the Zn was found in the fine fraction. These findings support strategies for efficient secondary use and recycling of FA, such as targeted Zn extraction from fine particles and potential utilization of the Ca-rich coarse particles in construction, reducing the reliance on virgin materials.

## Graphical abstract

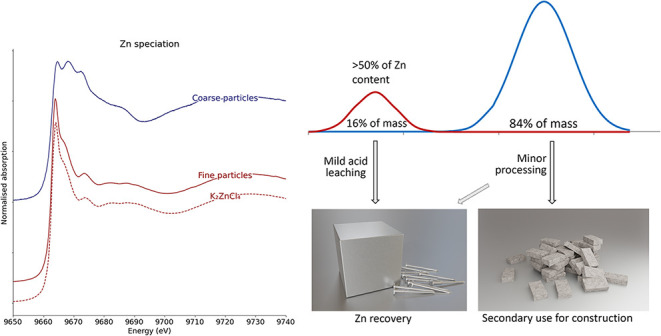

## 1 Introduction

Incineration is an efficient method for treating nonrecyclable waste, reducing its volume while recovering energy. The process generates solid residues, primarily bottom, and fly ash (FA). Potentially toxic metals such as Cd, Pb, and Zn are known to be enriched in FA.^1,2^ This raises environmental concerns, as metals may be mobilized from the ash, risking ecotoxic effects. Ecotoxicological reference values (ERVs) for metals such as Cr, Cu, Pb, and Zn are specified in several documents e.g.,^3^ from the European Chemicals Agency. The chronic ERV for all these metals is <20 μg/L, indicating the importance of preventing leaching to avoid potential ecotoxicological damage. Provided that the leaching of potentially toxic species is limited, FA may be utilized in construction.^4^

The presence of metals also offers opportunities for material recovery and recycling. It is particularly interesting to study the recovery of Zn, as Zn is relatively abundant in FA, ranging from 1 to 5 wt %.^1,5–8^ Main technologies for metal recovery from ash include hydrometallurgical and pyrometallurgical processes. The high energy demand and associated cost often limit the usefulness of pyrometallurgy methods. Instead, hydrometallurgical processes, where acid extracted from the flue gas cleaning is utilized as a leaching agent, have shown high Zn recovery rates (≥70%), and this method has recently been implemented at full scale.^9–12^

However, it is not only the concentration of toxic or valuable elements in FA that determines the environmental risk or recovery potential but also the chemical form of these. Understanding Zn speciation is therefore crucial for both efficient extraction in recovery processes and assessment of FA ecotoxicity. Earlier studies have employed X-ray diffraction (XRD) to investigate Zn speciation in waste-to-energy (WtE) FA. But due to the heterogeneous matrix, low Zn concentrations, and high amorphous content,^1,13^ XRD alone is insufficient.^14^ Scanning electron microscopy with energy dispersive X-ray spectroscopy (SEM-EDS) has also been applied,^14,15^ offering insights into both crystalline and amorphous phases. Despite this, the usefulness of SEM-EDS alone to determine speciation is limited, as it cannot confirm chemical bonding between colocated elements.

X-ray absorption spectroscopy (XAS), particularly X-ray absorption near edge structure (XANES), is one of the few techniques available for determining speciation of low-concentration metals (<1 wt %) in ash matrices.^16^ With XANES, the absorption edge of a specific element is analyzed, without interference from compounds that are not chemically bound to it. Using reference spectra and linear combination fitting (LCF), chemical forms of the target element can be identified. A number of studies have used XAS to investigate Zn speciation in WtE FA e.g.^17–20^ In a recent study on FA from five Nordic WtE facilities, Zn in reciprocating grate-boiler ash was predominantly present as alkali chlorides (∼50% of the Zn), with the remainder occurring as spinels, silicates, hydrozincite, and surface adsorbed Zn in varying proportions.^5^ Despite recent advances in understanding the Zn speciation in WtE FA by using XAS, knowledge remains limited. Analysis of XANES spectra is dependent on the inclusion of appropriate reference compounds, and LCF becomes more uncertain for heterogeneous mixes, which is the case of Zn compounds in ash. These issues together make Zn speciation challenging in practice.

In the high-temperature environment of the incinerator bed, noncombustible volatile elements evaporate and flow through the boiler with the flue gas. Condensates of these, together with solid material entrained from the combustion bed, form the FA.^15,21^ Nucleation and condensation of volatile species in the flue gas typically form fine particles (<1 μm aerodynamic diameter), whereas entrained particles are generally supermicron (>1 μm aerodynamic diameter), hereafter referred to as coarse. Zinc speciation is expected to differ between the respective size fractions due to the different formation mechanisms. Once FA is collected in air pollution control filters, the size-information is lost, as the surface forces acting on fine particles prevents effective redispersion^22^ and secondary reactions may change particle physicochemical state.

One previous study conducted size-selective in-flight sampling, directly from the flue gas channel, followed by XAS analysis on FA from WtE. Although a slight enrichment of zinc chlorides was observed in the finer particles, no clear differences in Zn speciation between fine and coarse fractions were reported.^17^ As no modal distribution of the fine and coarse mode particles was confirmed, this lack of differentiation could be due to the specific WtE facility design. Alternatively, it may stem from possible limitations in the sampling method, such as nonisokinetic sampling.

In this study, we investigate the Zn fractionation and speciation in fine and coarse fly ash particles, collected using size-separating particle sampling in-flight from the flue gas channel of an industrial WtE facility under isokinetic conditions. Two distinctly separated particle modes were confirmed with a minimum at ∼1–2 μm. By combining XANES with XRD, SEM-EDS, and elemental analysis on mode-specific samples, we aim to provide a more detailed understanding of Zn, allowing more efficient utilization of WtE FA.

## 2 Methods

### 2.1 Description of the Facility

All samples were collected from three grate-fired boilers at a full-scale WtE facility located in southwestern Sweden. The fuel feedstock consists of solid municipal and industrial waste, fed in proportions of about 1:1 throughout the year, except for the summer period when solely municipal waste is incinerated. The boilers were operating at full load, corresponding to 45 MW_th,el_ for boiler 1 and 54 MW_th,el_ for boiler 2 and 3.

Waste is fed onto a moving grate where air is constantly supplied both from underneath (primary) and above (secondary), ensuring that the temperature of the flue gas is kept above 850 °C (≥2 s) for complete combustion. Flue gas flows through the boiler, where it heats water to form high-pressure steam, powering a turbine. After passing through the economizer, particles are removed in an electrostatic precipitator (ESP). After the ESP, the flue gas passes through additional cleaning steps, including wet scrubbers.

### 2.2 Sample Collection

Samples were primarily collected at boiler 1, except for boiler ash samples that were also collected at boilers 2 and 3.

Different types of ash samples were collected: aerosol samples, collected “in-flight” by inserting a sampling probe into the flue gas channel, and “bulk” samples, here meaning ash deposits in the flue gas channel/in the boiler and in the downstream ESP filter. Using the in-flight sampling approach, samples containing ash particles of different size ranges can be collected before any major aggregation has occurred.

A more detailed description of the sample collection is found below, and an overview of the types of samples collected and their respective nomenclature is presented in Table 1. All samples were stored in sealed containers to limit moisture exposure and minimize chemical transformations. A schematic overview of the sampling points and further details are given elsewhere.^23^

**Table 1:** Overview of the Various Types of Samples Collected, and Sample Nomenclature^a^

sample name	sample description
*size-separated in-flight samples*	
coarse “X”	fly ash sample number “X” collected by cyclone (>1 μm), collected in parallel with fine “X”
fine “X”	fly ash sample number “X” collected on filter (<1 μm), collected in parallel with coarse “X”
Imp_D_50_	impactor sample where D_50_ indicates the cutoff of the impactor stage referred to (D_50_: 0.15/0.24/0.39/0.64/4.2/7.0/10.7 μm)
*bulk samples*	
ESP_winter/summer_	fly ash from ESP collected during winter and summer, respectively
BoAa “X”	boiler ash sample number “X” at position a (∼500–800 °C)
BoAb “X”	boiler ash sample number “X” at position b (∼200 °C)

a “X” is a sample running number for the respective sample type.

#### 2.2.1 In-Flight Collection of Size-Separated Fly Ash Particles

Ash samples were collected “in-flight” by sampling from the flue gas channel at a position just before the ESP, where the temperature of the gas is ∼220 °C. The sampling point is located upstream any flue gas cleaning processes. In-flight sample collection was performed with two different setups: while the impactor setup allows sampling in finer size intervals and was used to verify the bimodality of the particle size distribution, a cyclone-filter setup was used to achieve isokinetic conditions during sampling, and a separation of the fine and coarse mode particles. All cyclone-filter sampling and impactor sampling were performed at boiler 1. More details are found elsewhere^23^ and summarized below.

##### 2.2.1.1 Cyclone- and Filter Sampling

The cyclone-filter setup consists of a cyclone, collecting particles with diameters >1 μm, coupled in series with a filter, collecting all particles passing the cyclone, i.e., particles with a diameter <1 μm. Particles with diameters <1 and >1 μm are here referred to as the fine- and coarse mode particles, respectively. A “coarse” sample is always collected in parallel to a “fine” mode sample.

The cyclone was inserted into the flue gas channel and equilibrated with the temperature of the flue gas before collection. The filter holder was heated to above the dew point temperature of the flue gas (kept at 90 ± 10 °C). The sampling with the cyclone-filter setup was performed under isokinetic conditions. Two in-flight sampling campaigns were conducted in the same year, one in January and another in March. In total, 5 sample sets of fine and coarse particles were collected.

##### 2.2.1.2 Impactor Sampling

A low-pressure cascade impactor (DLPI, Dekati, Tampere, Finland) was used to collect FA separated into 13 size fractions ranging from 30 nm to ∼15 μm. The upper size was restricted by the fixed sampling flow to the DLPI (to achieve correct impactor stage cutoff), the 90° sampling angle to the flue gas flow, in combination with the particle velocity in the flue gas channel. This setup therefore did not capture the coarse mode particles fully. The samples collected using the impactor setup are named Imp_D_50_, where D_50_ refers to the cutoff (in μm) of the impactor stage, see Table 1.

#### 2.2.2 ESP Samples

Fly ash samples were collected from the ESP, directly from the hopper, in boiler 1. One sample was collected in the wintertime (MSW and industrial waste 1:1) and a second sample during the summer months, when burning only MSW.

#### 2.2.3 Boiler Ash Samples

The boiler ash samples (BoA) were collected at two positions: BoAa at an outlet directly after the first bend of the flue gas channel, where the flue gas cools from ∼800 to ∼500 °C, and BoAb further down the boiler at ∼200 °C. Boiler 1, where the in-flight samples and ESP samples were collected, only had one sampling outlet (BoAa) for boiler ash. To allow a comparison of boiler ash samples from different parts of the boiler, boiler ash samples were therefore also collected from two additional boilers placed at the same plant and burning the same fuel feedstock. In the analysis, distinction between the sample positions is made (a and b). However, since the boiler ashes were heterogeneous and showed no clear differences between boilers, these samples were not differentiated by boiler in the analysis.

### 2.3 Analyses

Samples were analyzed using XAS, SEM-EDS, and XRD, along with the determination of total elemental content. Not all samples were analyzed with all techniques due to practical aspects such as destruction, contamination during handling, and detection limits. A subset of samples of each type (listed in Table 1) was analyzed using each respective technique, when sample amounts allowed. An overview of the analyses performed on each sample is provided in Table S1.

#### 2.3.1 Total Elemental Content

The total elemental composition of the samples was determined by an accredited laboratory by analysis with inductively coupled plasma sector field mass spectrometry (ICP-SFMS) according to US EPA 200.8:1994, after digestion by fusion/HNO_3_, HCl, and HF. Method selection for the digestion was optimized depending on the sample matrix. For Cl analysis, samples were prepared by sintering at 550 °C with Na_2_CO_3_ and ZnO; water was leached and purified with a cation exchanger. Analyzed elements were Be, Na, Mg, Al, Si, P, S, Cl, K, Ca, Sc, Ti, V, Cr, Mn, Fe, Co, Ni, Cu, Zn, As, Sr, Y, Zr, Nb, Mo, Cd, Sn, Sb, Ba, La, W, Hg, and Pb.

For impactor substrates, the concentrations of many elements were below detection limits. Moreover, the elemental content at a specific stage could not be reliably linked to the total dry particle mass, as the substrates used for chemical analyses were not weighed gravimetrically. Weighing was avoided due to the risk of contamination and was also impractical for the thin, highly electrostatic substrates. Therefore, we do not present an elemental analysis for the impactor substrates.

#### 2.3.2 X-ray Diffraction

Coarse particles and ESP and boiler ash samples were ground and transferred to a standard powder sample holder. Impactor samples and fine mode particle samples were analyzed directly on the filters with a low-background Si sample holder. XRD measurements were performed using a Bruker D8 advance X-ray diffractometer (Cu Kα) equipped with a Lynxeye XE-T detector using a 2θ scan range of 10–80 °, increment 0.00458°/step, and 0.3 s/step.

Phase identification and Rietveld refinement for XRD data were performed using Profex (5.3.1). Fractions of crystalline and amorphous contents were estimated from automatic background fitting in DIFFRAC.EVA.

#### 2.3.3 Scanning Electron Microscopy

SEM-EDS measurements were performed in BSE mode using a Carl Zeiss EVO LS15 microscope equipped with an Oxford Instruments X-Max 80 mm^2^ detector. Samples were transferred to carbon tape before measurement.

#### 2.3.4 X-ray Absorption Spectroscopy

The Zn K-edge (9659 eV) of samples and reference compounds was measured at Balder beamline, MAX IV laboratory, using XAS on three occasions: ∼2 weeks after the first sample collection campaign, after ∼6 months, and after ∼1 year.

The monochromator energy was calibrated against a Zn reference foil, setting the derivative of the Zn K-edge (first peak) to 9659 eV. A ∼100 μm beam spot was used and an energy step size of 0.1 eV. To limit sample radiation damage, the beam position was slightly shifted in-between scans. Measurements were performed under ambient conditions and made in transmission mode to the largest extent possible. When not possible (e.g., impactor substrates, filter samples, and references with low Zn-content), either fluorescence data from a 7-element SDD detector or a large detector diode (PIPS) was used. The analysis was focused on the energy range of XANES.

##### 2.3.4.1 XAS Sample Preparation

References were ground and mixed with a polyethylene binder and pressed into pellets with 13 mm diameter, aiming at an optical thickness of ∼2. Ash collected on filters or impactor substrates was measured without prior preparation. Powder ash samples were in most cases, packed between two layers of Kapton tape, as tests showed that the sample preparation procedure involving pellet pressing might alter the chemical speciation of Zn. This resulted in a somewhat higher noise level due to the pinholes.

##### 2.3.4.2 XAS Data Treatment, Analysis, and Library of Zn-References

The spectra measured at different spots on the same sample were merged and normalized using the Bessy program (Freie Universität, Berlin, v.49). The XANES spectra were analyzed by linear combination fitting (LCF) with spectra of reference compounds. The fitting was performed using Athena (Demeter 0.9.26).^24^

Starting with all references, LCF was performed by removing all references one by one until only those above 5% were left. This was repeated several times, removing references in a different order to ensure consistency. The obtained group of compounds was used as a starting set for combinatoric calculations with a maximum of five components, since this was the minimum number of compounds needed for a satisfactory fit. The top 3–5 best fits from all combinations are discussed in the results.

A proper LCF for the identification and quantification of different compounds relies on the inclusion of relevant references to which the spectral signatures in the XANES spectra of the ash can be matched. Modeling of XANES spectra is yet uncertain, although there has been some progress in the area. Therefore, an important part of the study is the synthesis and inclusion of relevant references. The library of XANES reference spectra used in the analysis was extensive, comprising over 30 Zn-bearing compounds. A full list of included references is given in Supporting Information (SI), where XANES spectra of all references can be found (Figure S2 and data in SI). Most reference compounds have been used previously, described by Rissler and coauthors.^5^ The references synthesized specifically for the current study are Zn incorporated into crystalline and amorphous gehlenite (Zn_*x*+*y*_Ca_2‑*x*_Al_2‑*y*_SiO_7_), osakaite (Zn_4_(SO_4_)­(OH)_6_·5H_2_O), gordaite (NaZn_4_(SO_4_)­(OH)_6_Cl·6H_2_O), and Zn incorporated into gypsum (Zn_*x*_Ca_1–*x*_SO_4_·2H_2_O). Details about the synthesis and verification of these compounds are described in SI.

One reference synthesized and used also in our earlier study is KZnCl_3_. This compound is suggested in some fits of the fine mode particles in the current study. Notably, the crystal structure of this reference could not be confirmed by XRD as it was not present in accessible databases, but the ratio of K:Zn:Cl present matches KZnCl_3_, and the XRD spectrum of the reference was different from that of flinteite (K_2_ZnCl_4_) and other Zn-species, together indicating that the said compound was formed. The compound is known to contain crystalline water and, therefore, is hereafter denoted as KZnCl_3_·nH_2_O. The crystalline water is not expected to affect the XAS spectrum of Zn to any larger extent.

## 3 Results and Discussion

### 3.1 Size Separation of Fine and Coarse Particles and Elemental Content

In-flight sampling of FA from the flue gas channel revealed two distinct particle size modes, separated by a minimum around ∼1–2 μm.^23^ This confirmed that the cyclone-filter setup (with a cutoff of 1 μm) used in this study effectively separated the fine mode particles from the coarse mode particles. Gravimetric analysis of the cyclone-filter samples collected in parallel (coarse mode trapped in the cyclone and fine mode deposited on the filter) showed that the coarse mode particles accounted for on average 84% of the particle mass, while fine mode particles only 16% (presented in Table 2).

**Table 2:** Relative Fraction of Zn in Fine (<1 μm) and Coarse (>1 μm) Mode Particles, Presented for Individual Samples with Fine and Coarse Mode Particles and As Averages (±SD)^a^

	**fine particles**			**coarse particles**		
**cyclone-filter sample**	$\frac{\mathrm{Zn}\;\mathrm{mass}\;\mathrm{fine}\;\left(\mathrm{mg}\right)}{t\mathrm{otal}\;\mathrm{Zn}\;\left(\mathrm{mg}\right)}$	$\frac{\mathrm{Zn}\;\mathrm{mass}\;\mathrm{fine}\;\left(\mathrm{mg}\right)}{\mathrm{total}\;\mathrm{fine}\;\mathrm{mass}\;\left(\mathrm{kg}\right)}$	$\frac{f\mathrm{ine}\;\mathrm{mode}\;\mathrm{mass}\;\left(\mathrm{mg}\right)}{\mathrm{total}\;\mathrm{mass}\;\left(\mathrm{mg}\right)}$	$\frac{\mathrm{Zn}\;\mathrm{mass}\;\mathrm{coarse}\;(\mathrm{mg})}{t\mathrm{otal}\;\mathrm{Zn}\;\left(\mathrm{mg}\right)}$	$\frac{\mathrm{Zn}\;\mathrm{mass}\;\mathrm{coarse}\;\left(\mathrm{mg}\right)}{\mathrm{total}\;\mathrm{coarse}\;\mathrm{mass}\;\left(\mathrm{kg}\right)}$	$\frac{c\mathrm{oarse}\;\mathrm{mode}\;\mathrm{mass}\;\left(\mathrm{mg}\right)}{\mathrm{total}\;\mathrm{mass}\;\left(\mathrm{mg}\right)}$
**Fine1/Coarse1**	**0.55**	55,227	0.17	**0.45**	9280	0.83
**Fine2/Coarse2**	**0.45**	58,706	0.20	**0.55**	18,000	0.80
**Fine4/Coarse4**	**0.66**	100,198	0.16	**0.34**	9910	0.84
**Fine5/Coarse5**	**0.48**	80,233	0.13	**0.52**	12,800	0.87
**average**(±SD)	**0.54 ± 0.09**	73,591 ± 20,903	0.16 ± 0.03	**0.46 ± 0.09**	12,498 ± 3976	0.84 ± 0.03

a The paired fine and coarse samples (1, 2, 4, and 5) were collected in parallel using the cyclone-filter set-up. Also shown in the table are the data used for the calculation: Zn per dry ash mass in each mode (mg/kg), and the total mass fraction in each mode (from gravimetric analysis).

The elemental profiles differed markedly between the two modes; Cl, K, Na, and Zn were enriched in fine particles, whereas Ca, Si, and Al dominated in coarse particles. Interestingly, Zn was enriched in the fine mode, comprising 9% of the mass of the elements analyzed, compared to just 2% in the coarse mode. The average elemental composition of fine and coarse particles collected using the cyclone-filter setup is presented in Figure 1, given as wt % of the mass of elements analyzed by ICP-MS after digestion.

**Figure 1:**
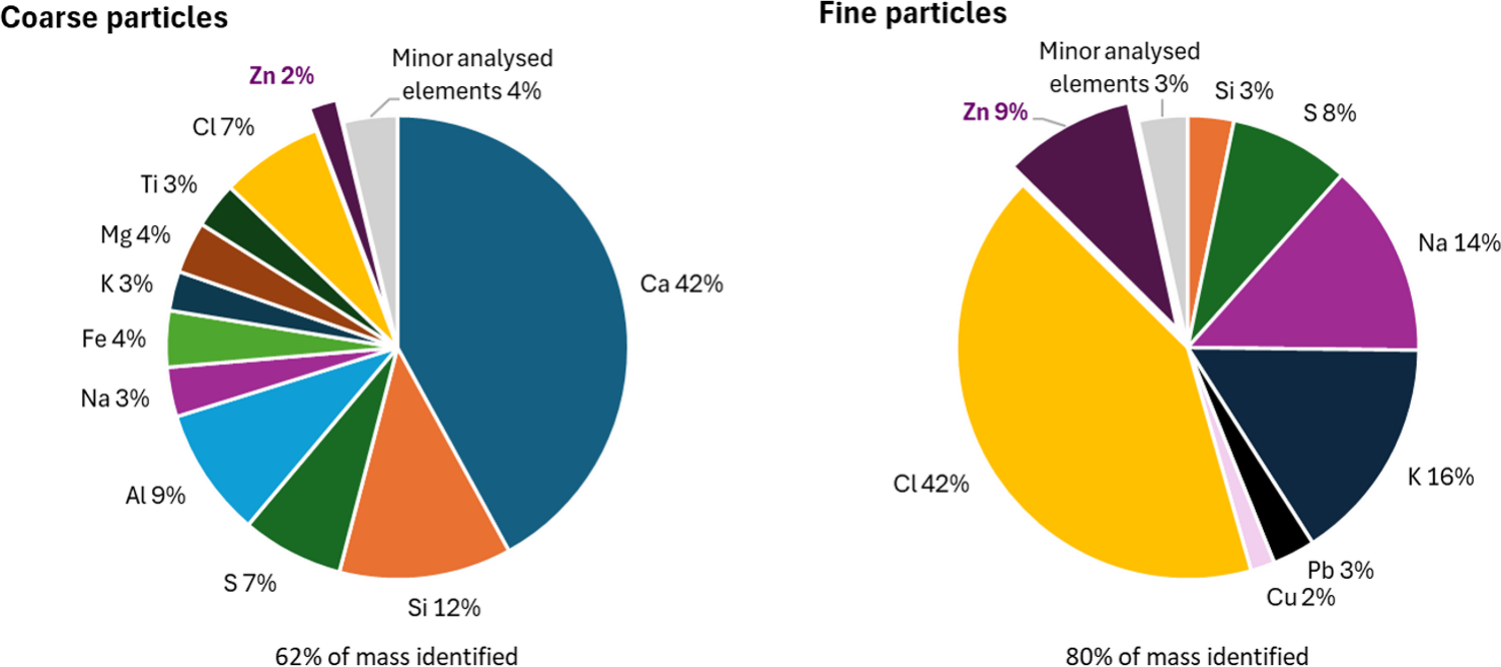
Major elemental composition of fine and coarse particles from the cyclone-filter in-flight sampling. The percentage values shown are based on the ICP-MS analyzed mass, i.e., elements such as C, O, and H are not included. Underneath each chart, the percentage of the total mass that could be identified (as any of the analyzed elements) is given. Only major elements (at least 2%) are shown, and identified elements in lower concentrations have been grouped as ‘minor analyzed elements’.

Based on the mass fraction (gravimetric) and the wt % Zn of ash dry mass in the respective mode, the fraction of Zn comprised in the fine mode was estimated according to

1 $${Zn}_{fine}=\frac{c_{fine}\cdot m_{fine}}{c_{fine}\cdot m_{fine}+c_{coarse}\cdot m_{coarse}}$$

where c_fine_ is the concentration (mg/kg dry particle mass) of Zn in the fine particle mode, m_fine_ the fraction of total ash mass in fine mode, c_coarse_ the Zn concentration (mg/kg dry particle mass) in the coarse particle mode, and m_coarse_ the fraction of total mass in the coarse mode. The Zn fraction in coarse mode particles is calculated as 1-Zn_fine_. The calculation revealed that Zn, on average, was close to equally distributed between the fine and coarse particles, varying between samples from 43 to 66% in the fine mode (presented in Table 2).

The elemental composition of the ash in the large-scale electrostatic filter (ESP_winter_ and ESP_summer_) showed that the ash was dominated by Ca, Na, K, S, and Si, as shown in Figure 2. Despite the seasonal difference in the fuel feedstock, the compositions of the two ESP samples were similar (Table S3). The boiler ash was dominated by Ca, Si, Al, and S, followed by Na, Fe, Mg, K, Ti, and Zn, also shown in Figure 2. The composition of the boiler ash sampled at positions a and b (BoAa and BoAb) was overall similar.

**Figure 2:**
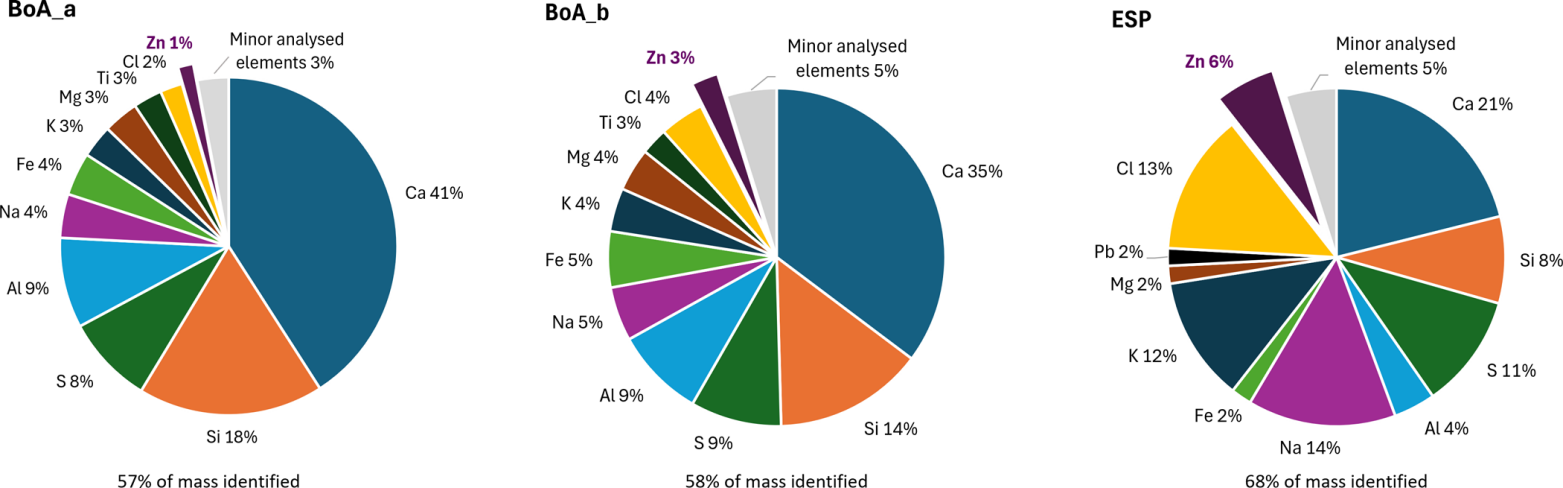
Major elemental composition of ESP and boiler ash (BoAa and BoAb). The percentage values shown are based on the ICP-MS-analyzed mass, i.e., elements such as C, O, and H are not included. Underneath each chart, the percentage of the total mass that could be identified (as any of the analyzed elements) is given. Only major elements (at least 2%) are shown, and identified elements in lower concentrations have been grouped as “minor analyzed elements”.

### 3.2 X-ray Diffraction

Identified crystalline components are listed in Table 3 and categorized by relative abundance into major (>30%), intermediate (10–30%), or minor (1–10%) components. Note that XRD only identifies crystalline phases, and a significant portion of the ash is known to be amorphous.^1,13^ The estimated amorphous fraction was estimated to be 48–58% (Table 3). This is similar to the amorphous fraction reported by Weibel et al.,^1^ which was approximately 40%.

**Table 3:** Crystalline Phases Identified by XRD^a^ [Table tbl1]

sample (estimated crystalline fraction)	major phase (>30%)	10–30%	minor phase (1–10%)
ESP_winter_ (54% )	halite NaCl	aphthitalite (K,Na)_3_Na(SO_4_)_2_	calcite CaCO_3_, **flinteite K**_**2**_ **ZnCl**_**4**_, anhydrite CaSO_4_, sylvite KCl, fluorite CaF_2_, periclase MgO, ulvöspinel Fe_2_TiO_4_, quartz SiO_2_
Fine1^b^	aphthitalite (K,Na)_3_Na(SO_4_)_2_, halite NaCl	**flinteite K**_**2**_ **ZnCl**_**4**_	
Fine2^b^	halite NaCl, aphthitalite (K,Na)_3_Na(SO_4_)_2_	**flinteite K**_**2**_ **ZnCl**_**4**_	
Coarse1 (48%)		anhydrite CaSO_4_, calcite CaCO_3_, gehlenite Ca_2_Al_2_SiO_7_, quarts SiO_2_	hematite Fe_2_O_3,_ halite NaCl, periclase MgO, sylvite KCl
Coarse2 (53%)		anhydrite CaSO_4_, quarts SiO_2_, calcite CaCO_3_, aluminum oxide Al_2_O_3_	bassanite CaSO_4_·0.5H_2_O, periclase MgO, halite NaCl, Al_17_Fe_8_Si_8_, sylvite KCl, quick lime CaO, fluorite CaF_2_
BoAa1 (56%)	calcite CaCO_3_	hydroxyapatite Ca_5_(PO_4_)_3_(OH)	aragonite CaCO_3_, syngenite K_2_Ca(SO_4_)_2_·H_2_O, gypsum CaSO4.2H2O, anhydrite CaSO_4_, perovskite CaTiO_3_, halite NaCl, Al_17_Fe_8_Si_8_, quartz SiO_2_, rutile TiO_2_
BoAa2 (59%)		hydroxyapatite Ca_5_(PO_4_)_3_(OH), calcite CaCO_3_, anhydrite CaSO_4_	gehlenite Ca_2_Al_2_SiO_7_, perovskite CaTiO3, periclase MgO, mayenite CaAl_2_O_3_, gypsum CaSO_4_·2H_2_O, halite NaCl, hematite Fe_2_O_3_, quartz SiO_2_
BoAb1 (48%)	calcite CaCO_3_	halite NaCl	hematite Fe_2_O_3_, aphthitalite (K,Na)_3_Na(SO_4_)_2_, quarts SiO_2_, mayenite CaAl_2_O_3_, anhydrite CaSO_4_, rutile TiO_2_, fluorite CaF_2_, sylvite KCl
Imp_0.39^b^,^c^	halite NaCl, sylvite KCl, aphthitalite (K,Na)_3_Na(SO_4_)_2,_ **flinteite K**_**2**_ **ZnCl**_**4**_		
Imp_4.2^b^,^c^	anhydrite CaSO4, halite NaCl, sylvite KCl, bassanite CaSO_4_·0.5H_2_O		
Imp_10.7^b^,^c^	halite NaCl, sylvite KCl, anhydrite CaSO4, aphthitalite (K,Na)_3_Na(SO_4_)_2_		

a The percentages correspond to the fraction of all crystalline compounds identified. Zn-containing phases are shown in bold. For an overview of sample types see Table 1.

b The crystalline fraction is not shown for all impactor and filter samples due to the sample substrate effects.

c Relative abundances are not given for impactor samples due to high noise level and difficult identification.

Only one Zn-containing compound was identified with XRD, namely, flinteite (K_2_ZnCl_4_), found in ESP ash and in all fine mode samples (Table 3). This and other identified peaks are showcased in the examples of XRD spectra from the ESP, fine, and coarse samples in Figure 3. The presence of K_2_ZnCl_4_ has been reported in MSWI fly ash samples in previous studies.^1,2,6,25^

**Figure 3:**
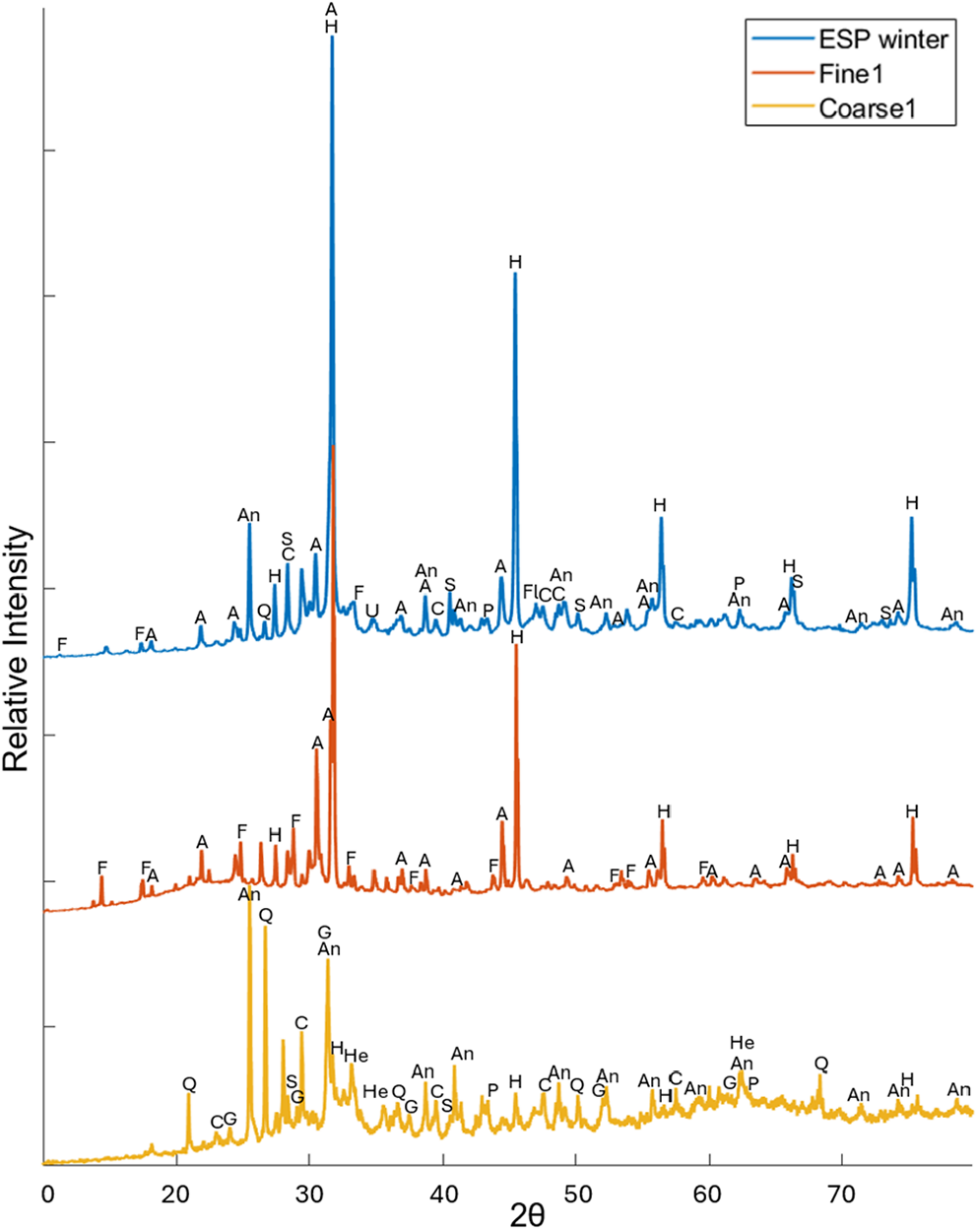
XRD spectra of the ESP ash, fine particles, and coarse mode particles. Flinteite (K_2_ZnCl_4_) is seen in ESP and Fine, but not in Coarse. The peaks are labeled with the corresponding phases and abbreviations are A: Aphthitalite (K,Na)_3_Na­(SO_4_)_2_, An: anhydrite CaSO_4_, C: calcite CaCO_3_, H: halite NaCl, S: sylvite KCl, F: flinteite K_2_ZnCl_4_, Fl: fluorite CaF_2_, P: periclase MgO, U: ulvöspinel Fe_2_TiO_4_, G: gehlenite Ca_2_Al_2_SiO_7_, Q: quarts SiO_2_, He: hematite.

Fine particles were typically more homogeneous compared to coarse particles in terms of the phases present. The phases in fine mode particles from the cyclone-filter setup were very similar to those identified in impactor stages corresponding to the fine mode. The most common phases found in the fine particles (<1 μm) were halite (NaCl) and sylvite (KCl). Aphthitalite ((K,Na)_3_Na­(SO_4_)_2_) was also identified in several fine samples, in ESP_winter_ and in BoAb1. The BoAb samples are deposits in the boiler, collected at a point in the boiler where flue gas temperature had decreased, and are therefore expected to contain some condensation products, in line with the fact that both aphthitalite, halite, and sylvite were identified in this sample.

The coarse and boiler ash samples were generally heterogeneous, containing a wide variety of crystalline phases, including many elements. No major (>30%) crystalline phase was identified in the coarse particle fraction. In the boiler ash, calcite (CaCO_3_) was identified as the only major compound. The most common phase in coarse particles was anhydrite (CaSO_4_), which was also present in boiler ash samples and in ESP ash. Other compounds identified in coarse mode were gehlenite (Ca_2_Al_2_SiO_7_) and hematite (Fe_2_O_3_). Quartz (SiO_2_) was also frequently observed in coarse particles. Overall, the compounds identified in this study (Table 3) are consistent with the XRD analysis of fly ash samples in previous studies.^1,2,6,25^

The BoAa samples, collected relatively close to the incineration bed, resembled the coarse particles, with the exception that hydroxyapatite (Ca_5_(PO_4_)_3_(OH)) was found only in BoAa. Hydroxyapatite has rarely been identified in FA in previous studies, but Bayuseno and Schmahl (2011) reported 2% apatite based on XRD.^25^

We report that about 50% of Zn is present in the coarse-mode particles. Despite this, XRD analysis of coarse and boiler ash samples did not identify any Zn phases. This is likely due to Zn being present as (i) solid solution in other crystalline phases, (ii) that Zn is present in amorphous forms, or (iii) that the concentration of Zn crystalline species is below the detection limit.

### 3.3 SEM-EDS

Focusing on Zn colocalization with other elements, SEM-EDS images were utilized for qualitative analysis. In fine mode particles, Zn was diffusely distributed in the samples, without distinct hotspots (Figure 4a). This was also the case in the coarse (cyclone-collected) particles, although some hotspots and heterogeneities were present (Figure 4b). In the impactor sample of coarse particles, some heterogeneity in Zn was observed, with spots of higher concentrations, but these could not be clearly correlated with any specific elements.

**Figure 4:**
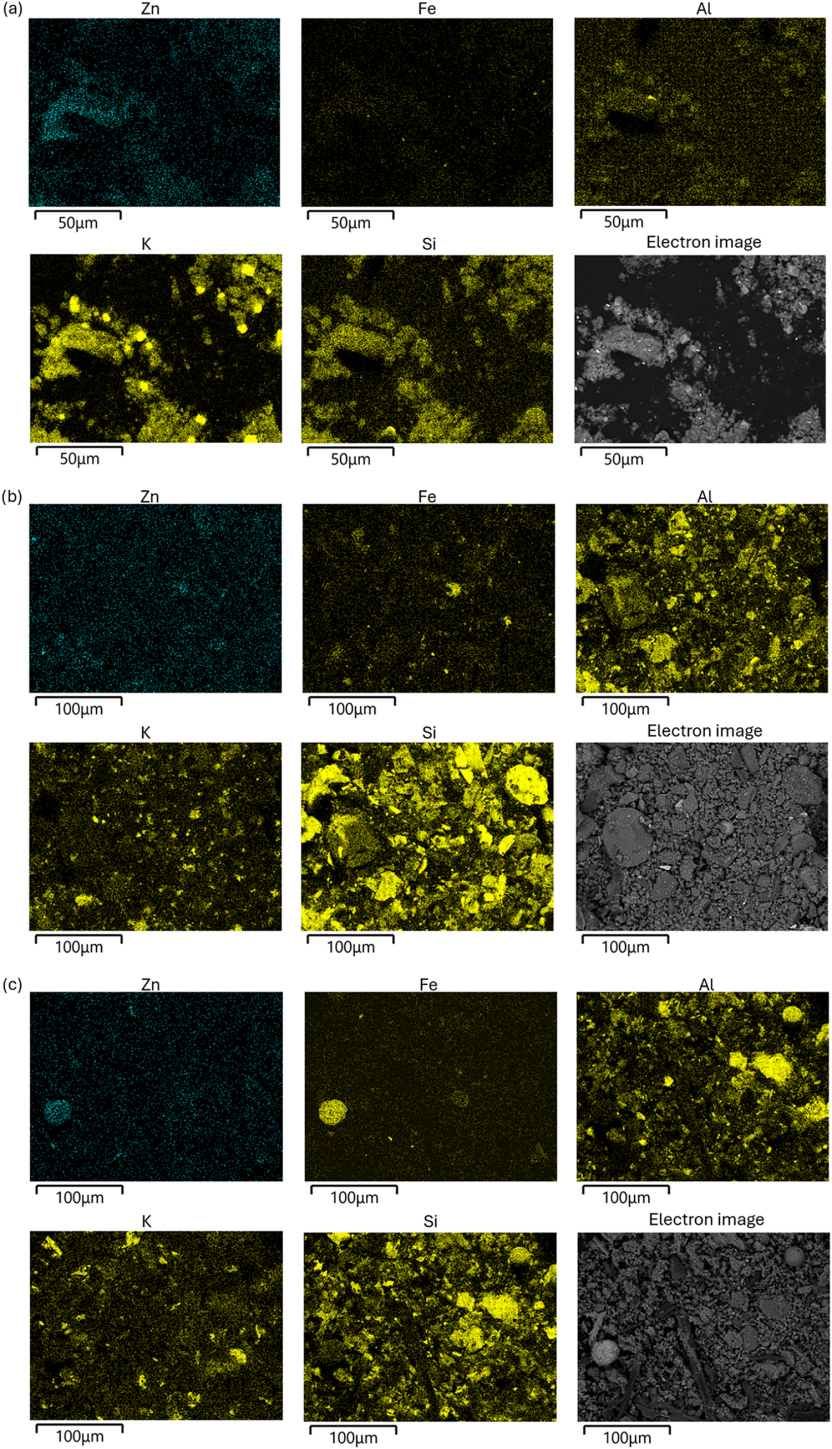
SEM-EDS images for samples (a) fine particles (Imp_0.39), (b) coarse particles (Coarse1), and (c) boiler ash (BoAa1) showing selected elements (Zn, Fe, Al, K and Si) along with the backscattered electron image.

In the boiler ash samples, there were many distinct large particles of different elemental composition, while Zn was again mostly diffusely distributed but with some exceptions. For example, in the BoAa1 image, there is a large spherical particle (to the left in Figure 4c) with high concentrations of Fe, Zn, and Mn. In the BoAb1 sample, there were several areas with hotspots of Zn, where some spots seemed to correlate with K and a few with Al (shown in Figure S1d). From visual inspection, it seems like there was less Zn in areas with high Ca concentration. For the ESP ash, the areas with the highest Zn concentrations correlated with Cl (Cl maps are shown in S1).

Almost everywhere in the samples Cl, Na, and S were present, but these elements were also observed in higher concentrations in certain areas (Figure S1). It seemed that Na and K were both colocated with Cl, which supports the XRD results where KCl and NaCl were identified. In a few particles, colocated Al and Si or Ca and S were seen, which supports identification of compounds such as gehlenite Ca_2_Al_2_SiO_7_, quartz SiO_2_, and anhydrite CaSO_4_ from XRD. In the analyzed regions of samples with coarse particles and ESP ash, occasional observations revealed Si-rich particles with little or no Ca. This indicates that Ca in these particles occurs in forms not associated with Si, such as CaSO_4_.

### 3.4 Zn Forms Determined by XAS

Results from the analysis of XANES spectra of the fine and coarse mode particles, collected using the cyclone-filter setup, revealed distinct differences in the chemical forms of Zn between the two modes (Figure 5).

**Figure 5:**
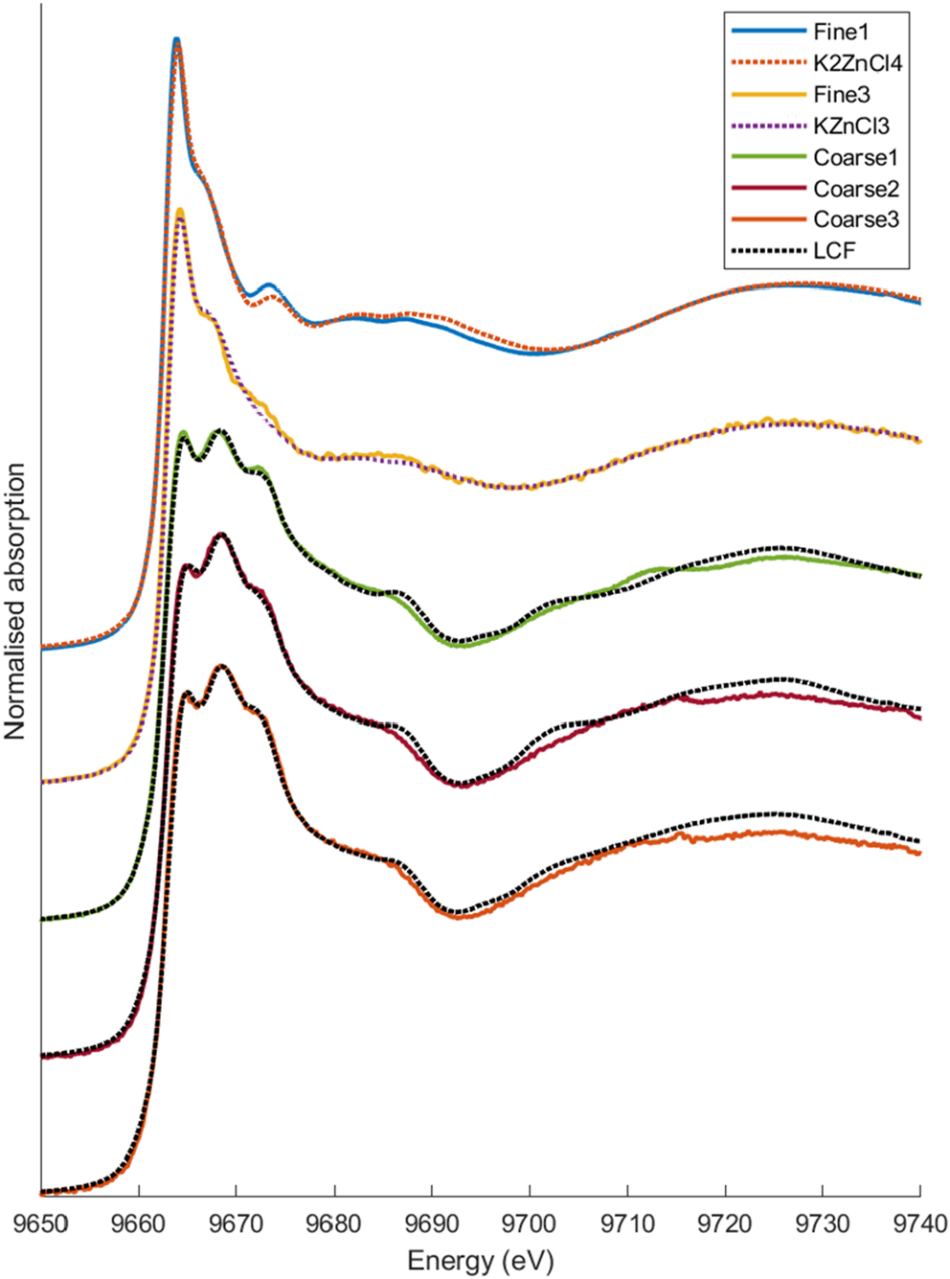
Representative XANES spectra at the Zn K-edge for the fine and coarse mode particles. According to LCF, Fine1 is dominated by K_2_ZnCl_4_, and Fine3 is dominated by KZnCl_3_·nH_2_O. Fine1 and 3 were chosen to represent these two distinct forms, and the corresponding reference is plotted together with the sample. For the coarse particles, the measured spectra are shown together with the results of LCF. The compounds found in the fits are listed in SI.

The spectra of the fine mode particles clearly showed that Zn was predominantly present as potassium zinc chlorides, specifically flinteite (K_2_ZnCl_4_) or cryobostryxite (KZnCl_3_·nH_2_O). In some samples, K_2_ZnCl_4_ was the dominant species according to the LCF analysis, while in others, KZnCl_3_·nH_2_O was more abundant. The Zn speciation for the fine mode samples estimated by LCF is given in SI, table S4. CaZnCl_4_ was excluded from the spectral fitting of the fine particles, since its formation via flue gas condensation has not been suggested in any previous studies, and Ca concentrations in these samples were very low (Figure 1). However, the CaZnCl_4_ spectral resemblance to K_2_ZnCl_4_ and KZnCl_3_·nH_2_O should be noted in relation to this.

The finding of K_2_ZnCl_4_ is supported by XRD (section 3.3), identifying it as a significant constituent in samples containing fine particles. Even though only one form of potassium zinc chloride salt was identified by XRD, it should be noted that the available databases (COD, ICSD) did not contain reference spectra for KZnCl_3_·nH_2_0.

Our findings are consistent with some previous observations on bulk ash samples collected from air pollution control systems, confirming the presence of K_2_ZnCl_4_ in the FA with XRD,^25,26^ and by XAS at the Zn-edge.^5^ Other previous XANES studies of FA did instead suggest Zn in the form of ZnCl_2_,^16,17,20^ and Struis and coauthors suggested hydrated ZnCl_2_ in fresh ash.^27^ However, these earlier XANES studies did not include K_2_ZnCl_4_ reference spectra. Studies using SEM-EDS have suggested the presence of K_2_ZnCl_4_ ^14^ or ZnCl_2_,^15^ based on the correlation between Zn and Cl. No earlier studies on fly ash from waste incineration suggest Zn in the form of KZnCl_3_·nH_2_0. In a study of minerals in active fumaroles, K_2_ZnCl_4_ is suggested to be unstable in a humid atmosphere, with KZnCl_3_·nH_2_O being the most common product.^28^ It is also possible that the small differences in XANES spectra found in the current results for the fine particles are due to disordered crystals of K_2_ZnCl_4_, and not due to the presence of KZnCl_3_·nH_2_0.

The sulfur-to-chloride ratio is known to influence the Zn-products formed, as high SO_2_ concentrations in flue gas can inhibit the formation of zinc chlorides.^17,29^ In the current study, the Cl:S molar ratio is around 5:1 for the fine mode particles, where nearly all Zn is found in the form of potassium zinc chloride. Thus, our results are in line with the theory, although Zn is not found as ZnCl_2_ as often suggested, but as K_*x*_ZnCl_*y*_. For the coarse mode particles and thus also for the ESP ash, the Cl:S molar ratio is lower (for coarse mode particles ∼0.8). The coarse mode originates from entrainment of bottom ash and is primarily composed of refractory Zn forms, with some condensates.

Typically, the Zn XANES spectra of the fine mode samples collected at the second campaign were more similar to those of KZnCl_3_·nH_2_O than those collected at the first campaign. The samples collected at the second occasion were analyzed six months postcollection (compared to 2 weeks for the first set of samples), due to limited access to XAS beamtime. One hypothesis was therefore that the observed difference was caused by chemical transformations during storage. In an attempt to investigate whether there were any indications of consistent transformations over time, the samples were reanalyzed after one year of storage, also including a subset that had been deliberately exposed to high humidity. The results did not provide conclusive evidence of systematic changes in Zn speciation over time into KZnCl_3_·nH_2_O (Supporting Information, Table S4). Both forms of potassium zinc chloride were present in the samples. Instead, the results suggested that the variation stems from differences between sampling occasions and variation across sampling spots (beam spot size ∼100 μm) on the filters.

In previous work, it was demonstrated that Zn in fly ash from air pollution control systems can transform from K_2_ZnCl_4_ into simonkolleite (Zn_5_(OH)_8_Cl_2_·H_2_O) upon exposure to ambient humidity during storage.^5^ In the same study, it was shown that the typical XAS sample preparation methods, such as grinding and tablet pressing, accelerate such transformation. Samples of fine particles exposed to humidity and remeasured after 12 months of storage showed a very slight increase in the estimated fraction of Zn_5_(OH)_8_Cl_2_·H_2_O, but still below 5% (Table S4). Simonkolleite was also identified as a minor Zn-species in one sample that was measured only after ∼12 months of storage (Fine4), accounting for approximately 15% of the Zn. Thus, it seems that the transformation of K_2_ZnCl_4_ into Zn_5_(OH)_8_Cl_2_·H_2_O is not occurring for the size-separated samples to the same extent as in the mixed ESP ash, containing all particle sizes. This may indicate that the transformation requires a higher pH, where the ESP ash is expected to be more alkaline due to the high Ca content from coarse particles.

Coarse particle Zn speciation was heterogeneous, meaning that there was no single dominant form of Zn, in contrast to the fine particles. This is in line with earlier XANES results for ESP ash samples.^5,19^ Three distinct peaks were present in the Zn-spectra of coarse mode particles, corresponding to the typical spectral features of Zn-aluminate and Zn-ferrites (ZnAl_2_O_4_, ZnFe_2_O_4_). The five best results from LCF, each limited to five Zn compounds, suggest that Zn in the coarse mode particles was in the form of Zn dissolved into amorphous gehlenite (Zn_*x*+*y*_Ca_2‑*x*_Al_2‑*y*_SiO_7_) (25–40%), Zn-aluminate (ZnAl_2_O_4_) (15–23%), and zinc adsorbed/incorporated into ferrihydrite (Zn_Fhy) (12–41%). The range in parentheses represents the typical variation in the five best fits. Additional compounds suggested by the fits were zinc chlorides (ZnCl_2_ or K_2_ZnCl_4_), Zn-ferrite (ZnFe_2_O_4_), hydrozincite (Zn_5_(CO_3_)_2_(OH)_6_) and alkaline Zn-sulfates (NaZn_4_(SO_4_)­(OH)_6_Cl or Zn_4_(OH)_6_SO_4_).

The presence of Zn dissolved into spinel phases in FA (represented by ZnAl_2_O_4_ and ZnFe_2_O_4_, both with similar spectral features) is supported by the SEM-EDS for BoAa1, where a large particle with colocations of Zn, Mn, and Fe was seen. The observation further suggests that Zn is not the only element incorporated into the ferrites but that other transition metals could be involved, forming mixed-metal spinels such as (Mn_*x*_Zn_*y*_)­Fe_2_O_4_.

To quantify Zn associated with the coarse versus fine modes within the ESP samples, which are in some aspect more representative of ash collected over longer periods, one approach is to fit the XANES spectra of the ESP ash using the spectra of the fine and coarse mode particles. The XANES spectra of the ESP samples are shown in Figure 6 together with LCF. For ESP_winter_, the fit suggests that about 40% of the Zn is associated with the fine fraction. An even better fit was obtained when simonkolleite (Zn_5_(OH)_8_Cl_2_·H_2_O) was added, resulting in a fit where 46% of the Zn is represented by Fine1, 45% by Coarse1, and 9% simonkolleite. By including simonkolleite in the fine mode, the estimated fractionation between Zn associated with the fine mode was the same as that determined from the in-flight sampling (54% in both cases; see Figure 6 and average in Table 2). This suggests that some Zn (K_2_ZnCl_4_/ KZnCl_3_·nH_2_O) from fine particles in ESP ash is transformed to simonkolleite during storage, as previously suggested.^5^

**Figure 6:**
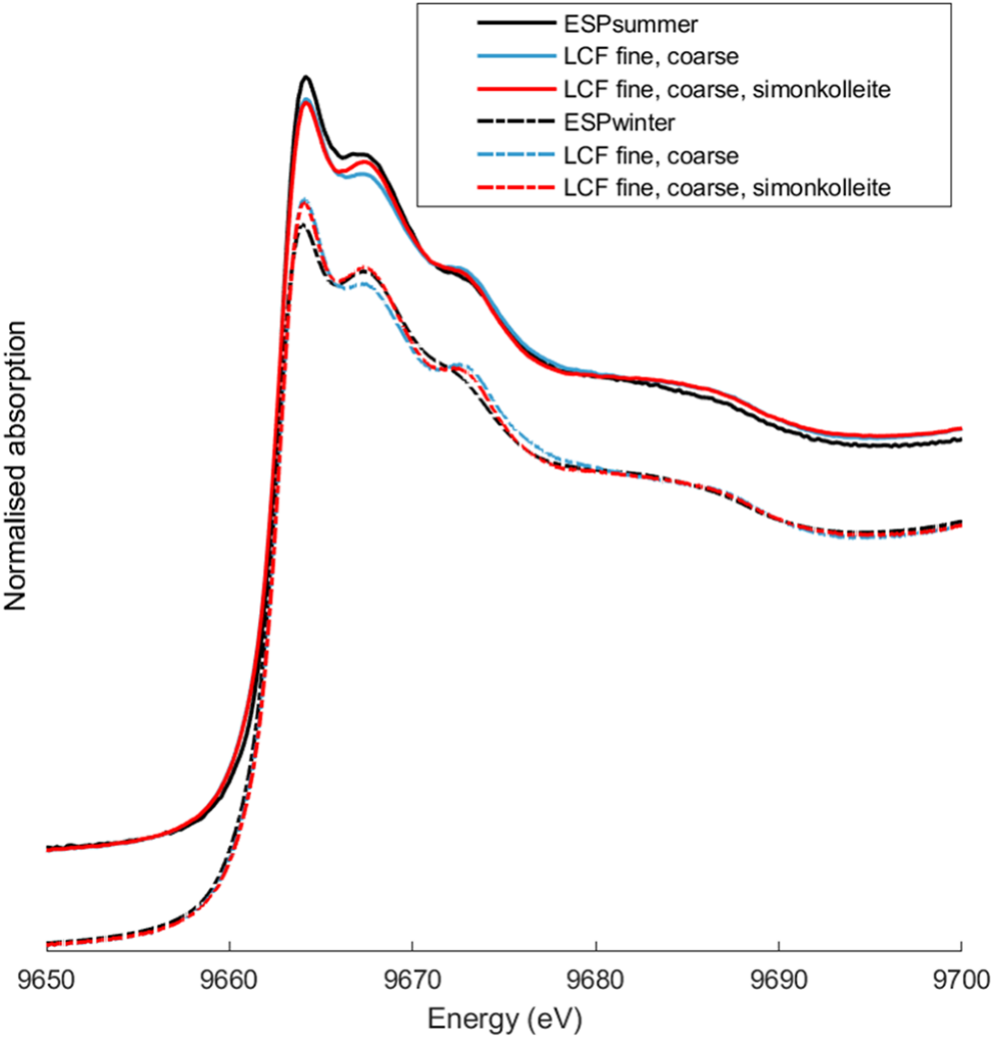
XANES spectra at the Zn-edge for the two ESP samples (solid lines summer, dashed lines winter) and corresponding spectra reconstructed by fitting the spectra with XANES spectra of fine and coarse mode (blue lines) and when adding also simonkolleite (Zn_5_(OH)_8_Cl_2_·H_2_O) spectra (red).

The LCF for ESP_summer_ indicated a slightly higher portion of Zn in the fine mode of 65% (58% fine, 7% simonkolleite, and 35% coarse). This value (65%) is closer to the fraction of Zn earlier reported in the form of alkali chlorides (typically associated with the fine mode) in ESP ash from the same facility.^5,30^ The variation in the Zn partitioning between fine and coarse mode particles may be due to several factors, including differences in fuel feedstock or fluctuations in primary air flow, which can affect the amount of ash entrained from the combustion bed to the flue gas. For ESP_winter_, the fitting was done using XANES spectra of fine and coarse mode particles collected at the same season as the ESP_winter_ ash, while for the summer sample, the average of all fine and coarse spectra, respectively, was used, as no in-flight sampling was performed during summer. This slight difference in approach did not cause the higher suggested fraction of Zn in the fine mode for ESP_summer_.

Using the full library of references, the LCF analysis of the ESP FA (ESP_winter_ and ESP_summer_) was repeated to see if results were consistent with the species from coarse and fine particle LCF. For both ESP samples, the main compounds in the three best fits were Zn doped into amorphous gehlenite (Zn_*x*+*y*_Ca_2‑*x*_Al_2‑*y*_SiO_7_), Zn-aluminate (ZnAl_2_O_4_), and chloride salts (K_2_ZnCl_4_, ZnCl_2_, or KZnCl_3_·nH_2_O). Additionally, Zn-ferrite (ZnFe_2_O_4_) was suggested in the ESP_Summer_ sample and hydrozincite (Zn_5_(CO_3_)_2_(OH)_6_) in ESP_Winter_. In several fits, a minor fraction of Zn-doped gypsum (Zn_*x*_Ca_1–*x*_SO_4_·2H_2_O) was also appearing. Overall, these results are in agreement with the LCF results for the fine and coarse fractions. However, the fitting approach becomes less certain the more complex mix of Zn-species, as would be expected when mixing the condensation- and entrained particles that together make up the ESP FA.

Impactor samples showed a similar trend in their XANES spectra as the samples from the filter and cyclone setup, with stages collecting the smallest particles resembling the fine mode and stages collecting the larger particles resembling the spectra of the coarse mode (Figure 7). All samples except for the Imp_4.2 could be fitted well with the spectra of samples Fine1 and Coarse1. This was the only sample that deviated from the gradual trend in Zn speciation, observed as a function of the D_50_ of the impactor stages. A possible explanation could be that impactor samples containing coarse particles are more susceptible to variability in composition due to short sampling times and few particles in the analyzed area.

**Figure 7:**
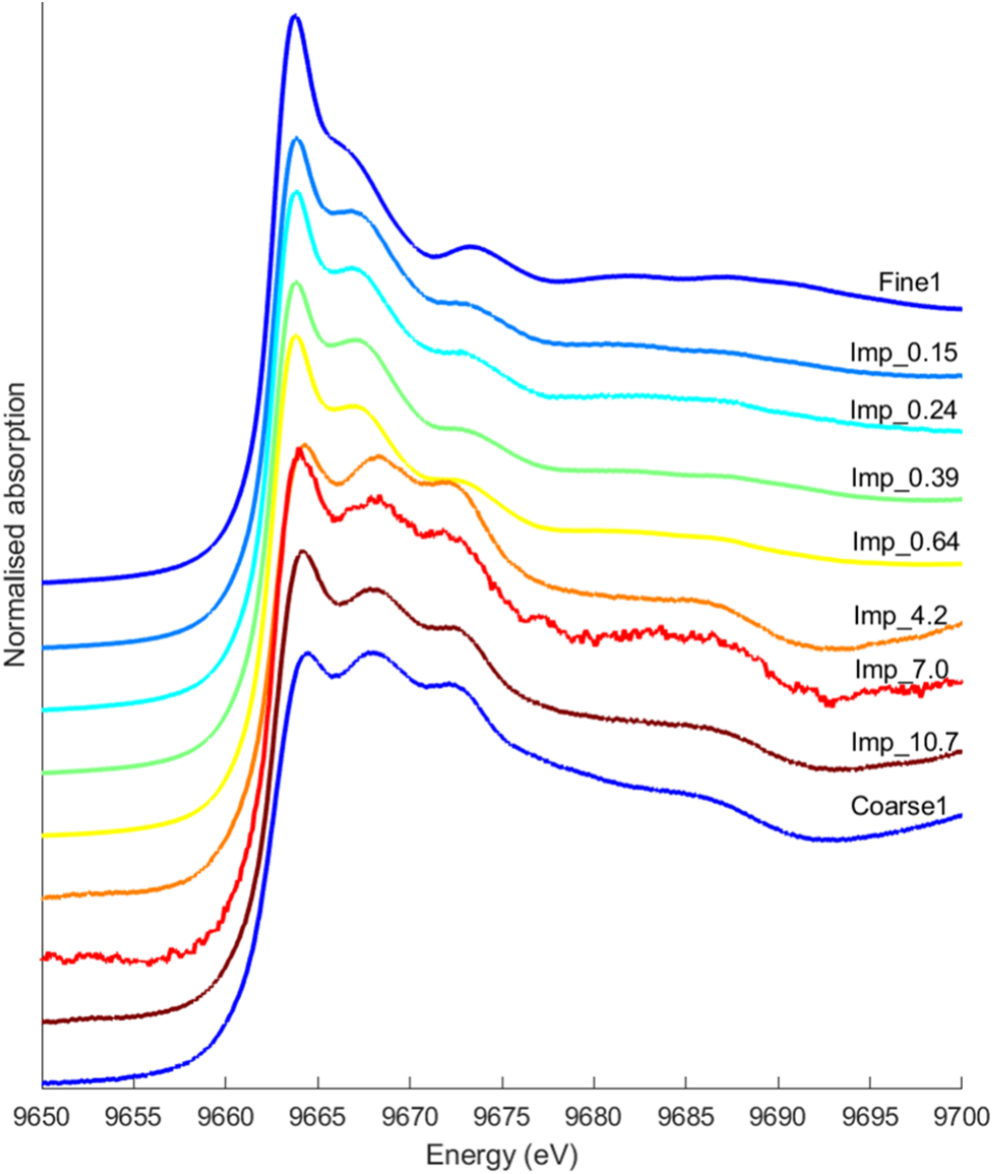
Average Zn XANES spectra of impactor samples from different impactor stages, where the number corresponds to the upper cutoff of the stage (D_50_) in μm. Fine and coarse mode spectra from the cyclone-filter setup are shown for comparison.

The impactor XANES spectra confirmed that Zn speciation varies over particle size although the differentiation between the fine and coarse mode particles was not as distinct as from the cyclone-filter setup but showed a more gradual change in Zn form. This could be an indication that coarse particles are entrained into the lower impactor stages by particle bounce, which is often a result of overloading the impactor. Furthermore, the coarse particles collected by the impactor are not fully representative of the entire coarse mode as the sampling was not performed isokinetically, as in the case using a cyclone-filter setup. Other factors to consider when comparing XAS spectra from the two set-ups are that the samples collected by the cyclone-filter setup had longer sampling times (more representative) and were better protected against moisture during storage.

Ash deposited in the boiler (BoAa and BoAb) revealed substantial heterogeneity in Zn forms, both between individual XAS measurement spots within a sample and between samples collected on different occasions. For the final analysis, all XANES spectra for each sample type (BoAa and BoAb) were averaged (Figure 8). Individual spectra are provided in the Figure S3 and Supporting Information (SI).

**Figure 8:**
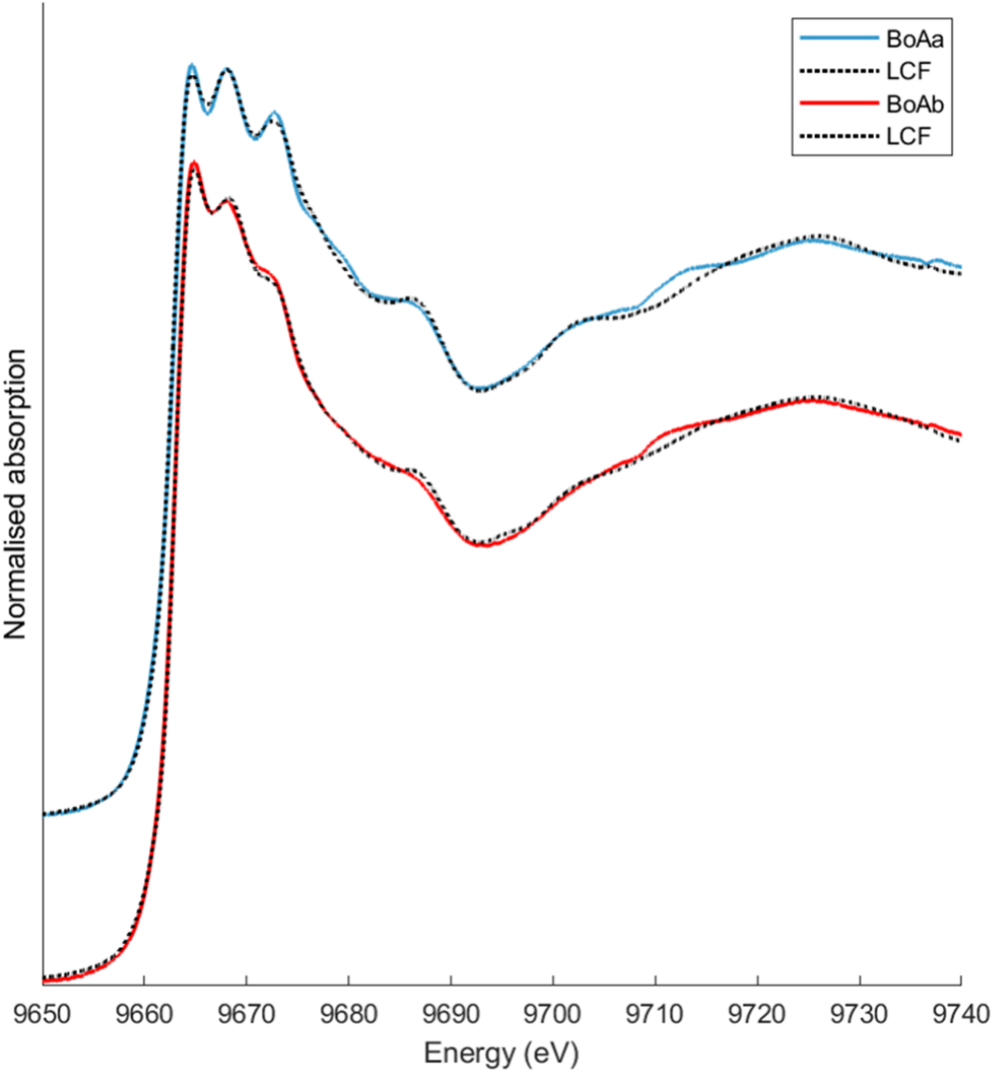
Average Zn K-edge XANES spectra for BoAa and BoAb samples, along with the corresponding LCF. The individual spectra here averaged are given in SI, Figure S3, along with details about the LCF.

The LCF indicated that BoAa contained a highly heterogeneous mix of Zn compounds, including silicate in the form of Zn-doped gehlenite (Zn_*x*+*y*_Ca_2‑*x*_Al_2‑*y*_SiO_7_) in mostly amorphous but also crystalline form_,_ spinels (ZnAl_2_O_4_, ZnFe_2_O_4_), minor amounts of chlorides, and either hopeite (Zn_3_(PO_4_)_2_·4H_2_O) or Zn adsorbed onto ferrihydrite. The Zn composition in BoAb was similar to that of BoAa, but with a higher proportion of Zn chlorides. In other words, Zn speciation in BoAa (closest to the incinerator) more resembled the coarse-mode particles, while BoAb (collected further downstream, where the flue gas was ∼200 °C) showed characteristics of both particle modes. This aligns with expectations that particles at point “a” are primarily refractory materials entrained from the combustion bed but too large (aerodynamically) to follow the flue gas through the channel bend. At point “b”, lower temperatures have partly promoted the formation of condensational products in the flue gas channel, and the deposits contain both entrained particles and condensates, as also indicated by the XRD results.

Zinc dissolved in glass was observed in BoAb, and in one fit, also Zn-doped gypsum (Zn_*x*_Ca_1–*x*_SO_4_·2H_2_O) was suggested. The presence of a Zn-doped glassy silicate phase and possibly Zn-doped gypsum in boiler FA is strengthened by the identification of quartz (SiO_2_) and anhydrite (CaSO_4_) by XRD.

Zinc incorporated into crystalline gehlenite (Zn_*x*+*y*_Ca_2‑*x*_Al_2‑*y*_SiO_7_) was only suggested from the fit of BoAa. Interestingly, this is consistent with that crystalline gehlenite was identified by XRD in the same specific sample, supporting the conclusion that Zn can indeed be incorporated into the gehlenite structure in fly ash. In other ash samples, including coarse and ESP ash samples, Zn was only associated with amorphous gehlenite (i.e. not detectable by XRD). To our knowledge, no previous study has shown that Zn in FA occurs dissolved into gehlenite (as a solid solution). However, Zn incorporation into gehlenite has been described previously,^31^ and gehlenite has been identified in fly ash^1^ from WtE processes. It has also been shown that during heat treatment of ash, oxide minerals can transform into gehlenite.^32^

#### 3.4.1 Uncertainties in XANES Interpretation

In the complex heterogeneous ash mixtures present in coarse mode particles, several reference compounds are interchangeable in the LCF, resulting in similar R-values in the fit. This is because they have spectral features with close resemblance, making it difficult to unambiguously identify individual Zn-species. Examples of such compounds are silicates (e.g., Zn-doped amorphous gehlenite vs Zn-doped glass), spinels (ZnAl_2_O_4_ vs ZnFe_2_O_4_, and to some extent Zn adsorbed onto ferrihydrite), chloride salts (K_2_ZnCl_4_ vs CaZnCl_4_, and KZnCl_3_·nH_2_O), and sulfate-containing compounds (gordaite, osakaite, simonkolleite, and Zn-doped gypsum). Additionally, Zn-doped glass and Zn-doped gypsum appear to be replacing each other in the LCF, despite the difference in the local chemical environment of Zn.

Besides the challenges associated with complex mixtures and reference compounds with similar spectral features, fitting accuracy is influenced by factors such as energy calibration, normalization parameters, and the energy range fitted; i.e., careful result interpretation and use of complementary methods are warranted. When using the same fitting approach as in our earlier study,^5^ but with the extended library of references, we could also see varying results from LCF when changing the order of the references included in the fitting software. XAS is a powerful tool in identifying speciation of low-content elements in complex samples. However, the heterogeneous nature of coarse mode particles underscores the importance of combining multiple analytical techniques (e.g., XAS, XRD, and SEM-EDS) and careful spectral fitting to improve confidence in phase identification. One promising approach to improve the XANES analysis and identification of Zn species in heterogeneous ash is the use of two-dimensional XANES mapping. This approach provides spatially resolved information within the sample and allows for the identification of localized regions or particles that contain more pure phases. EXAFS can also serve as a complementary technique to distinguish between chemical forms and provide additional insights into compounds with similar XANES features, for example, by analyzing first- and second-nearest neighbor distances.

## 4 Conclusions and Implications

A primary objective of this study was to investigate the distribution and speciation of Zn in fine and coarse mode FA particles from WtE, based on the knowledge that the speciation is largely determined by particle formation and transformation mechanisms. To do so, particles were collected “in-flight” from the flue gas channel using size-specific sampling.

We demonstrate that over 50% of the Zn is found in fine mode particles (<1 μm) of WtE FA, and Zn is present almost exclusively in the form of highly soluble potassium zinc chloride salts. A more complex mixture of Zn compounds is found in coarse mode particles and boiler ash, dominated by refractory and less leachable forms such as spinels, other ferrites, and silicates like gehlenite.

Our findings could be used to facilitate more efficient secondary use and material recycling of FA. For example, introducing in-flight separation of fine and coarse particles (e.g., a cyclone) at an industrial scale could enable highly efficient Zn extraction from fine FA particles by leaching. The Zn speciation of the coarse particle mode showed a high proportion of stable Zn forms (silicates and spinels). This suggests that this Ca-rich fraction could potentially be used more safely (compared to the ESP ash) in construction as a substitute for virgin materials, provided that other potentially toxic elements, such as Cu, also occur in stable forms.

## Supplementary Material





## Notes

Additional experimental details for the syntheses of reference materials, full list of XAS reference compounds and XAS spectra of all references, linear combination fitting results for fine particles, all individual XAS spectra for boiler ash samples (DOCX)
An excel file with raw data: normalized XANES spectra for ash samples, normalized XANES spectra for reference compounds, XRD spectra for ash samples (XLSX)
