# Supplementary material for: Zinc Speciation in Fine and Coarse Fly Ash Particles Collected In-Flight at a Waste Incinerator
Source: ACS Environ Au. 2026 Jan 30;6(2):283–94. doi: 10.1021/acsenvironau.5c00222 (PMC13003353; doi:10.1021/acsenvironau.5c00222)
Supplement: Supplementary file 1 [file vg5c00222_si_001.docx]

**Supporting Information**

**Zinc speciation in fine and coarse fly ash particles collected in-flight at a waste incinerator** Evelina Gorjatšova^‡1^, Fanny Bergman^‡1^, Kajsa G. V. Sigfridsson Clauss^2^, Nils Skoglund^3^, Karin Karlfeldt Fedje^4,5^, Jenny Rissler^1,6*^

^1^ Ergonomics and Aerosol Technology, Department of Design Sciences, Lund University, SE-22100 Lund, Sweden, 
^2^ MAX IV Laboratory, Lund University, Box 118, SE-22100 Lund, Sweden

^3^ Thermochemical Energy Conversion Laboratory, Department of Applied Physics and Electronics, Umeå University, SE-901 87 Umeå, Sweden

^4^ Recycling and Waste Management, Renova AB, Box 156, SE-401 22 Gothenburg, Sweden

^5^ Department of Architecture and Civil Engineering, Chalmers University of Technology, SE-412 96 Gothenburg, Sweden

^6^ NanoLund, Lund University, SE-22100 Lund Sweden

^‡^ Splitted 1^st^ authorship due to equal contributions.

^*^ Corresponding author, jenny.rissler@design.lth.se

**Table S1.** Detailed information on sample naming, collection occasion, substrate type and characterization methods.

| **Sample name** | **Sample description** | **Characterisation** | **Collection occasion, substrate** |
| --- | --- | --- | --- |
| Bulk samples | | |  |
| ESP_Winter_ | Fly ash from ESP of boiler 1 collected during winter | ICP-MS, XAS (2 weeks after collection), XRD, SEM-EDS | January |
| ESP_Summer_ | Fly ash from ESP of boiler 1 collected during summer | ICP-MS(soon), XAS (9 months after collection) | July |
| BoAa1 | Boiler ash from boiler 1, position a, sample 1 | ICP-MS, XAS (2 weeks after collection), XRD, SEM-EDS | January |
| BoAa2 | Boiler ash from boiler 3, position a, sample 2 | ICP-MS, XAS (2 weeks after collection) | January |
| BoAa3 | Boiler ash from boiler 1, position a, sample 2 | ICP-MS, XAS (2 weeks after collection) | January |
| BoAa4 | Boiler ash from boiler 2, position a, sample 1 | XAS (2 weeks after collection) | January |
| BoAa5 | Boiler ash from boiler 2, position a, sample 2 | XAS (2 weeks after collection) | January |
| BoAa6 | Boiler ash from boiler 3, position a, sample 1 | ICP-MS, XAS (2 weeks after collection), XRD | January |
| BoAb1 | Boiler ash from boiler 3, position b, sample 1 | ICP-MS, XAS (2 weeks after collection), XRD, SEM-EDS | January |
| BoAb2 | Boiler ash from boiler 2, position b, sample 2 | XAS (2 weeks after collection) | January |
| BoAb3 | Boiler ash from boiler 3, position b, sample 2 | ICP-MS, XAS (2 weeks after collection) | January |
| In-situ sampling | | |  |
| Coarse1 | FA collected in cyclone (>1 µm), sample 1 | ICP-MS, XAS (2 weeks after collection), XRD, SEM-EDS | January |
| Coarse2 | FA collected in cyclone (>1 µm), sample 2 | ICP-MS, XAS (6 months after collection), XRD, SEM-EDS | March |
| Coarse3 | FA collected in cyclone (>1 µm), sample 3 | ICP-MS, XAS (6 months after collection) | March |
| Coarse4 | FA collected in cyclone (>1 µm), sample 4 | ICP-MS | March |
| Coarse5 | FA collected in cyclone (>1 µm), sample 5 | ICP-MS | March |
| Fine1 | FA (<1 µm) collected on filter in parallel with Coarse_1 | ICP-MS, XAS (2 weeks and 1 year after collection), XRD, SEM-EDS | January, quartz |
| Fine2 | FA (<1 µm) collected on filter in parallel with Coarse_2 | ICP-MS, XAS (6 months and 1 year after collection), XRD, SEM-EDS | March, quartz |
| Fine3 | FA (<1 µm) collected on filter in parallel with Coarse_3 | XAS (6 months after collection) | March, polycarbonate |
| Fine4 | FA (<1 µm) collected on filter in parallel with Coarse_4 | ICP-MS, XAS (1 year after collection) | March, quartz |
| Fine5 | FA (<1 µm) collected on filter in parallel with Coarse_5 | ICP-MS | March, polycarbonate |
| ImpE_0.15 | Impactor sample E, stage >0.15 µm | XAS (2 weeks after collection) | January, polycarbonate |
| ImpE_0.24 | Impactor sample E, stage >0.24 µm | XAS (2 weeks after collection) | January, polycarbonate |
| ImpE_0.39 | Impactor sample E, stage >0.39 µm | XAS (2 weeks after collection), XRD | January, polycarbonate |
| ImpE_0.64 | Impactor sample E, stage >0.64 µm | XAS (2 weeks after collection) | January, polycarbonate |
| ImpE_4.2 | Impactor sample E, stage >4.2 µm | XAS (2 weeks after collection) | January, polycarbonate |
| ImpE_7.0 | Impactor sample E, stage >7.0 µm | XAS (2 weeks after collection) | January, polycarbonate |
| ImpE_10.7 | Impactor sample E, stage >10.7 µm | XAS, (2 weeks after collection), XRD | January, polycarbonate |
| ImpG_0.15 | Impactor sample G, stage >0.15 µm | XAS (2 weeks after collection) | January, polycarbonate |
| ImpG_0.39 | Impactor sample G, stage >0.39 µm | XAS (2 weeks after collection), XRD | January, polycarbonate |
| ImpG_10.7 | Impactor sample G, stage >10.7 µm | XRD | January, polycarbonate |
| ImpD_4.2 | Impactor sample D, stage >4.2 µm | XRD, SEM-EDS | January, aluminium |
| ImpD_10.7 | Impactor sample D, stage >10.7 µm | XAS (2 weeks after collection) | January, aluminium |
| ImpF_0.39 | Impactor sample F, stage >0.39 µm | XRD, SEM-EDS | January, polycarbonate |
| ImpF_10.7 | Impactor sample F, stage >10.7 µm | XRD, SEM-EDS | January, polycarbonate |
| ImpH_0.39 | Impactor sample H, stage >0.39 µm | XRD | January, polycarbonate |
| ImpH_10.7 | Impactor sample H, stage >10.7 µm | XRD | January, polycarbonate |

**Synthesis and verification of reference compounds**

The purity of the reference materials was verified using XRD, with additional confirmation in some cases by SEM-EDS. References were verified using STOE Stadi MP X-ray diffractometer (Cu K𝛼) using scan range 10-60 2Theta. Zn doped gehlenite was measured using 0.45°/step, 5 s/step and other references using 0.105°/step, 5 s/step.

Osakaite (Zn_4_(OH)_6_SO_4_^.^5H_2_O) was synthesised using a procedure adapted from (Moezzi et al., 2013). In brief, 33 ml 0.1M sodium hydroxide (NaOH) aqueous solution was added to 30 ml 0.1M zinc sulphate heptahydrate (ZnSO_4_^.^7H2O) aqueous solution slowly while stirring. The mixture was stirred for 1 hour at room temperature, then left to settle for 1 hour and centrifuged 10 min. Precipitate was decanted, filtered using 0.22 µm bottle top filter and washed three times with water. Compound was dried for three days in a desiccator at room temperature.

Gordaite (NaZn_4_(OH)_6_(SO_4_)Cl·6H_2_O) was synthesised using zinc oxide hydrolysis method as described in literature (Leal et al., 2020; Machado Silva & Wypych, 2022; Maruyama et al., 2017). 50 ml of aqueous solution containing 7.5 mmol zinc sulphate heptahydrate (ZnSO_4_·7H2O) and 22.5 mmol sodium chloride (NaCl) were added to the beaker containing 19.8 mmol zinc oxide (ZnO). The beaker was closed with parafilm and stirred for 168 hours at room temperature. The mixture was left to settle for 90 min, after which it was centrifuged 7 min. The precipitate was decanted and resuspended in 50 ml water by putting in the ultrasonic bath for a few seconds. Washing was repeated five times. The compound was dried for three days in a desiccator at room temperature.

Zinc-doped gypsum was synthesised by titration of calcium hydroxide with sulfuric acid. 3.1 mmol zinc sulphate heptahydrate (ZnSO_4_·7H_2_O) were dissolved in 50 ml 1M sulfuric acid (H_2_SO_4_). Acid solution was added dropwise to the mixture of 061 mol calcium hydroxide (Ca(OH)_2_) in 34 ml deionized water, while measuring the pH. Mixture was left to stabilise for 1 hour and pH was adjusted to 6.95. Pure gypsum was prepared in a similar way without addition of zinc sulphate, with final pH of 7.16. The precipitate was filtered, washed with water and dried overnight at 40°C. 5g of Zn-doped gypsum were then added to 40 ml saturated pure gypsum solution in 0.5M HCl and filtered. Compound was washed 4 more times with acidified saturated gypsum solution, washed 2 times with water and dried for 6 hours at 40°C. XRD data confirmed that the samples were pure gypsum and no other zinc species was identified. In SEM-EDS for this sample no Zn hotspots were seen, while in the sample with higher Zn concentration bright crystals containing Zn were present. This could be interpreted as that the synthesis with lower Zn concentration was successfully resulting in a solid solution of Zn (Zn replacing Ca in the crystal). However, no change in the lattice distances were observed, possibly explained by the low concentration of Zn. Even though Zn adsorbed to gypsum surfaces could not be ruled out, we judge the likeliness low as the samples were wash with acid after formation.

Microbeads of Zinc-doped gehlenite (Ca_2_Al[AlSiO_7_) was synthesised by combining a solid-state reaction of the precursor powders, followed by flame synthesis. The synthesis followed the procedure described in the study by (Majerová et al., 2019), replacing Ni with Zn. Samples of four different Zn-concentrations were prepared: 0% (undoped), 0.5%, 1% and 4% (mol %) according to the table below.

**Table S2.** Elemental composition of the Zn-doped gehlenite prepared (mol. %).

|  | CaO | Al_2_O_3_ | SiO_2_ | ZnO |
| --- | --- | --- | --- | --- |
| GZn0 | 50 | 25 | 25 | 0 |
| GZn1 | 49.25 | 24.87 | 24.88 | 0.5 |
| GZn2 | 49.5 | 24.75 | 24.75 | 1 |
| GZn3 | 48.5 | 24.25 | 24.25 | 3 |

The prepared samples were amorphous (by XRD). A subsample was treated by heat to determine the crystallisation temperature. The crystalline material formed was also analysed by XRD and XAS, where XRD conformed gehlenite. The XAS spectra for the lowest concentration of Zn was used in the analysis as this was argued being most relevant. The 0.5% and 1% crystalline references had similar XAS spectra while the reference with 3% had a somewhat different spectrum, all amorphous spectra were the same.

**Table S3.** Total elemental content of ESP samples given in mg/kg dry mass.

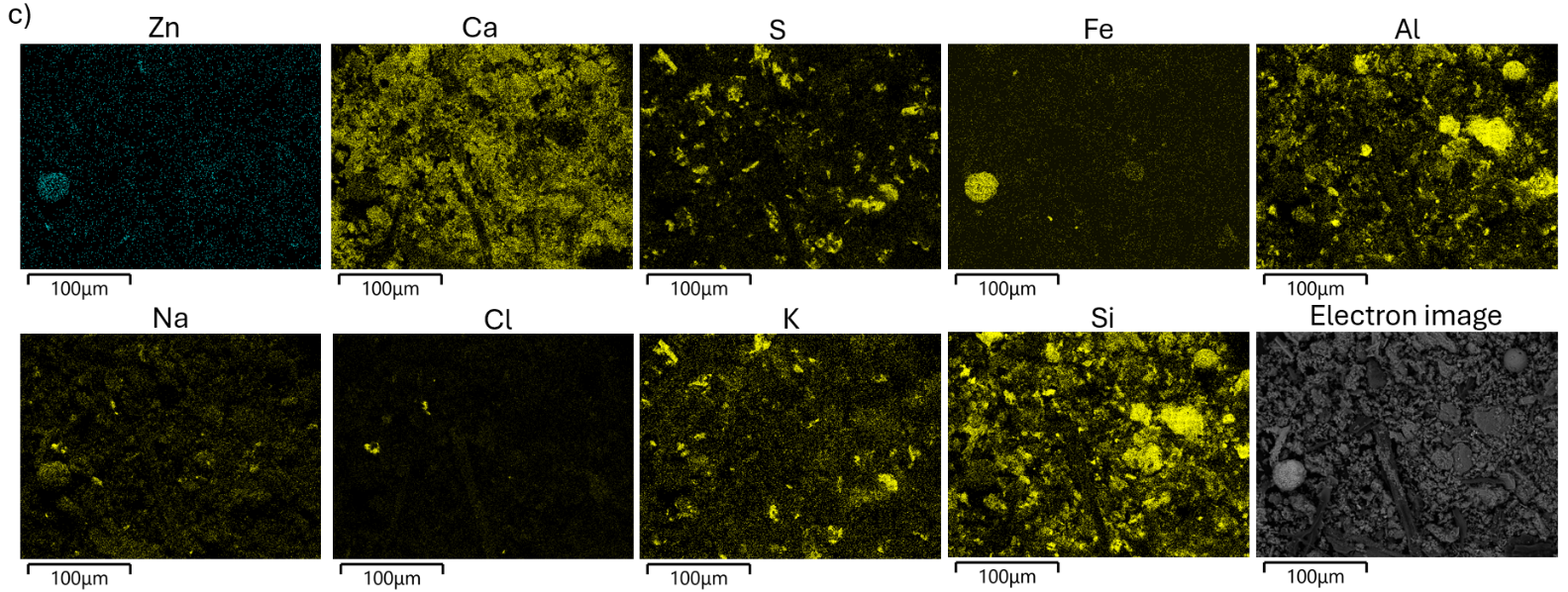

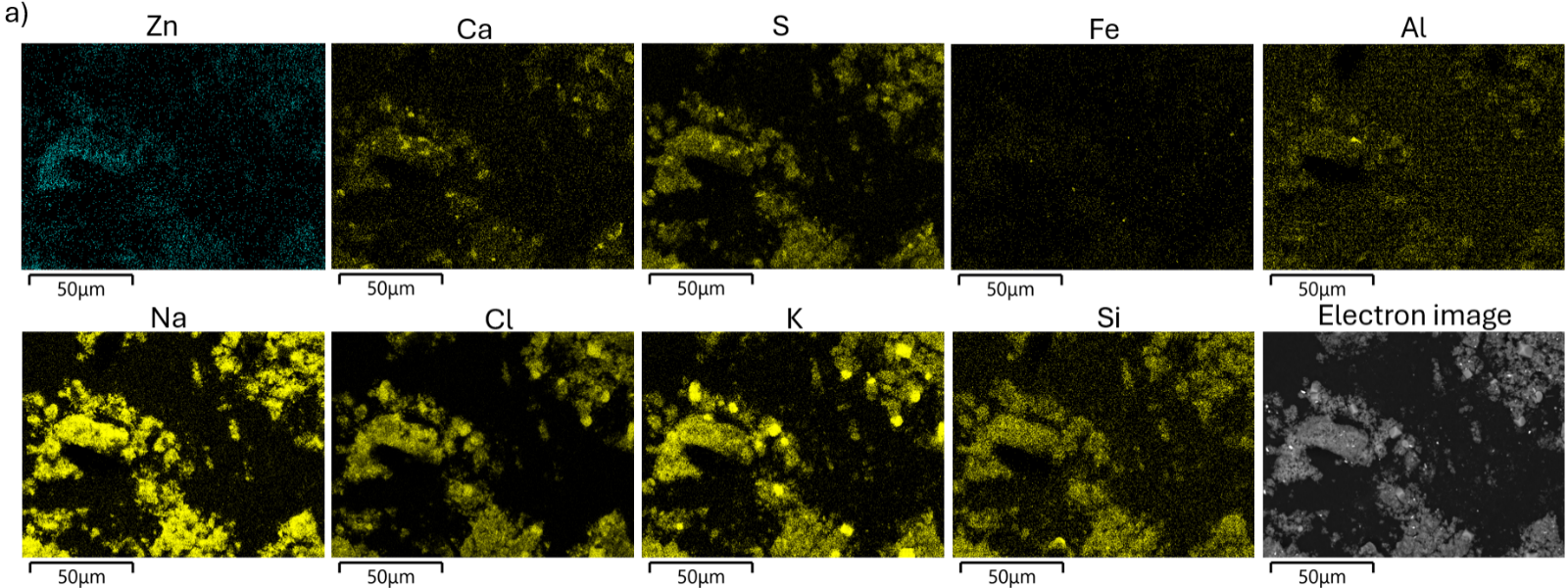

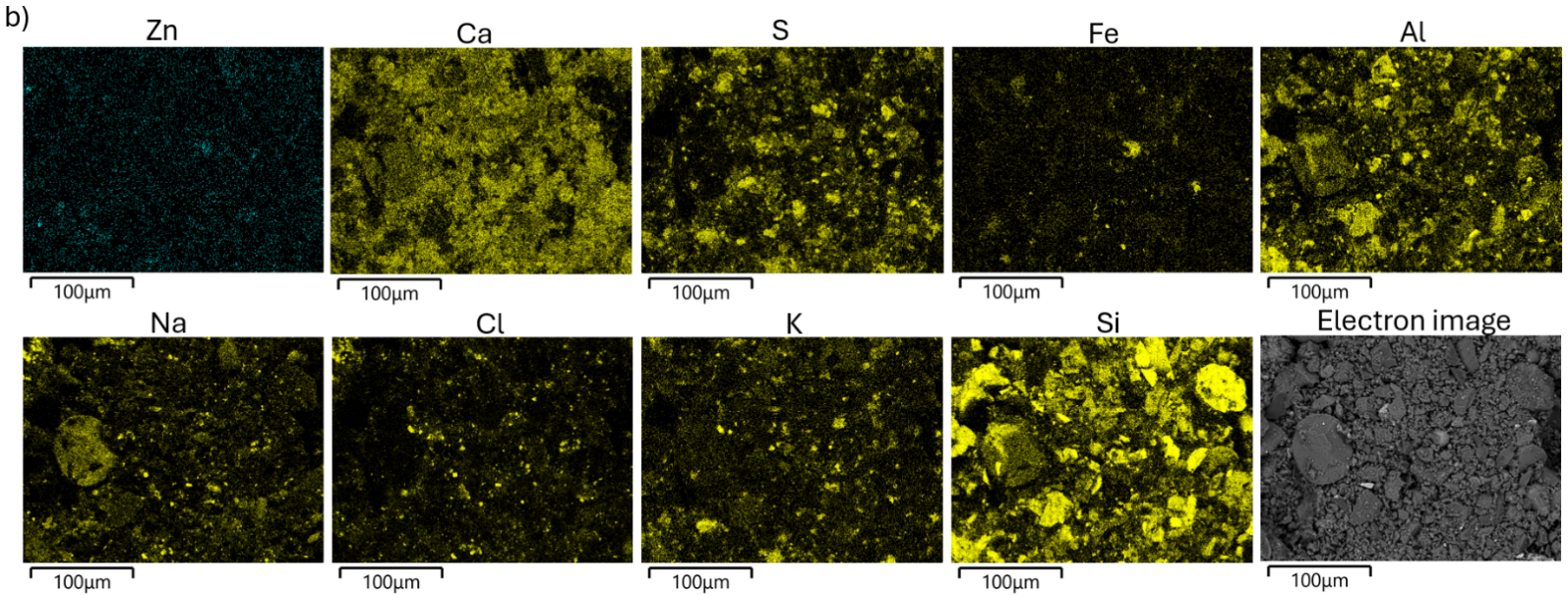


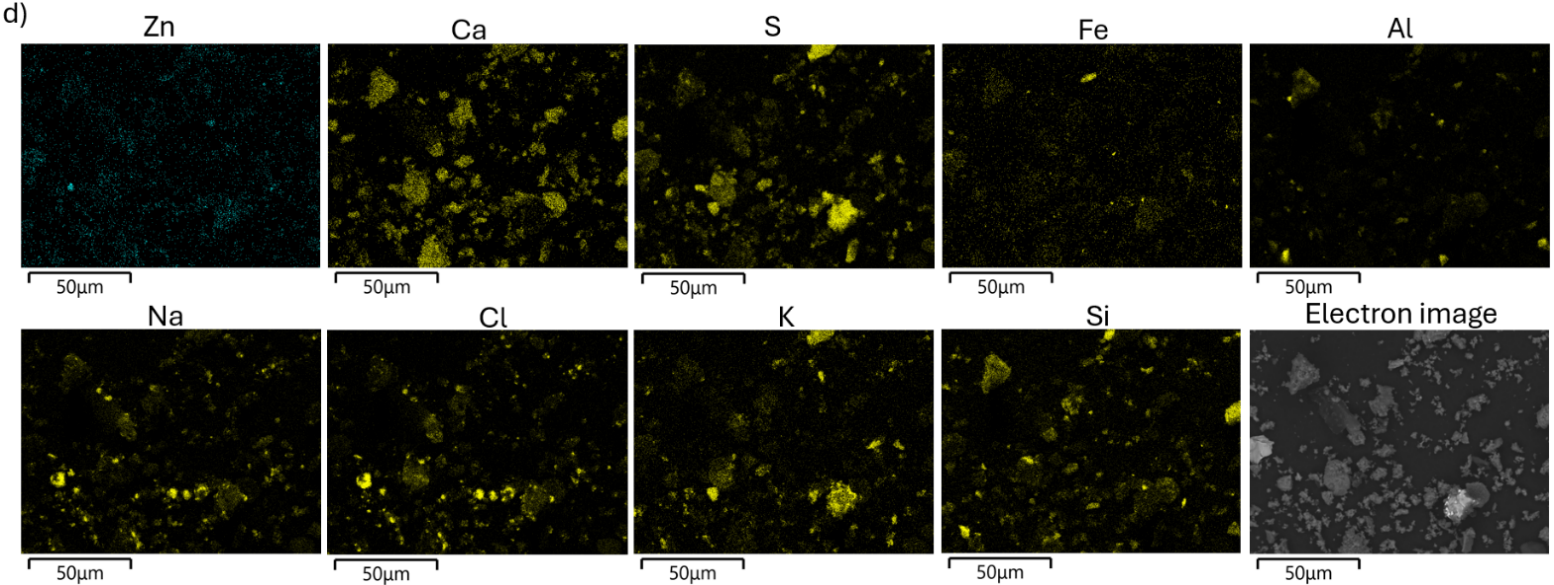
**Figure S1**. SEM-EDS images for samples a) fine particles (Imp_0.39), b) coarse particles (Coarse1), c) boiler ash (BoAa1), d) boiler ash (BoAb1) showing selected elements (Zn, Ca, S, Fe, Al, Na, K and Si) along with backscattered electron image.


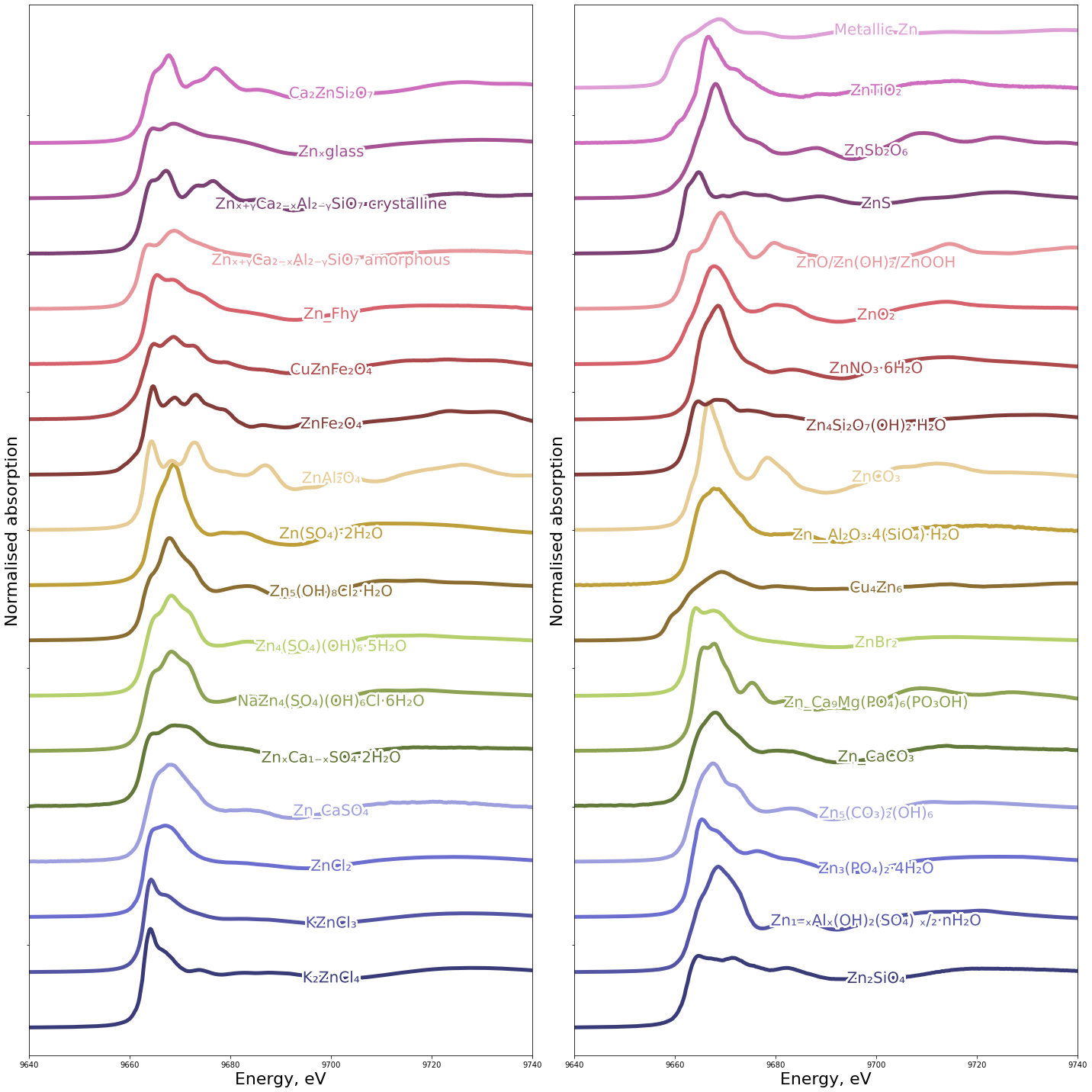

**Figure S2**. Reference spectra Zn K-edge. All references used: metallic Zn, hardystonite (Ca_2_ZnSi_2_O_7_), Zn incorporated into glass (Zn_x_glass), Zn adsorbed to the surface of ferrihydrite Fe_5_O_3_(OH)_9_ (Zn_Fhy), Zn adsorbed to the surface of calcium sulphate (Zn_CaSO_4_), Zn adsorbed to the surface of bentonite (Zn_Al_2_O_3_·4SiO_4_·H_2_O), simonkolleite (Zn_5_(OH)_8_Cl_2_·H_2_O), hemimorphite (Zn_4_Si_2_O_7_(OH)_2_·H_2_O), copper zinc ferrite (CuZnFe_2_O_4_), zinc ferrite (ZnFe_2_O_4_), zinc aluminate (ZnAl_2_O_4_), zinc sulphate (ZnSO_4_·2H_2_O), zinc chloride (ZnCl_2_), zinc titanate (ZnTiO_2_), zinc antimonate (ZnSb_2_O_6_), zinc sulphide (ZnS), zinc oxide (ZnO/Zn(OH)_2_/ZnOOH), zinc dioxide (ZnO_2_), zinc nitrate (ZnNO_3_·6H_2_O), zinc carbonate (ZnCO_3_), brass (Cu_4_Zn_6_), zinc bromide (ZnBr_2_), Zn adsorbed to whitlockite (Zn_Ca_9_Mg(PO_4_)_6_(PO_3_OH)), Zn adsorbed to calcium carbonate (Zn_CaCO_3_), hydrozincite (Zn_5_(CO_3_)_2_(OH)_6_), hopeite (Zn_3_(PO_4_)_2_·4H_2_O), zincowoodwardite (Zn_(1-x)_Al_x_(OH)_2_[SO_4_]_x/2_·nH2O), willemite (Zn₂SiO₄), flinteite (K_2_ZnCl_4_), cryobostryxite (KZnCl_3_·nH_2_O), Zn incorporated into crystalline and amorphous gehlenite (Zn_x+y_Ca_2-x_Al_2-y_SiO_7_), osakaite (Zn_4_(SO_4_)(OH)_6_·5H_2_O), gordaite (NaZn_4_(SO_4_)(OH)_6_Cl·6H_2_O), Zn incorporated into gypsum (Zn_x_Ca_1-x_SO_4_·2H_2_O).


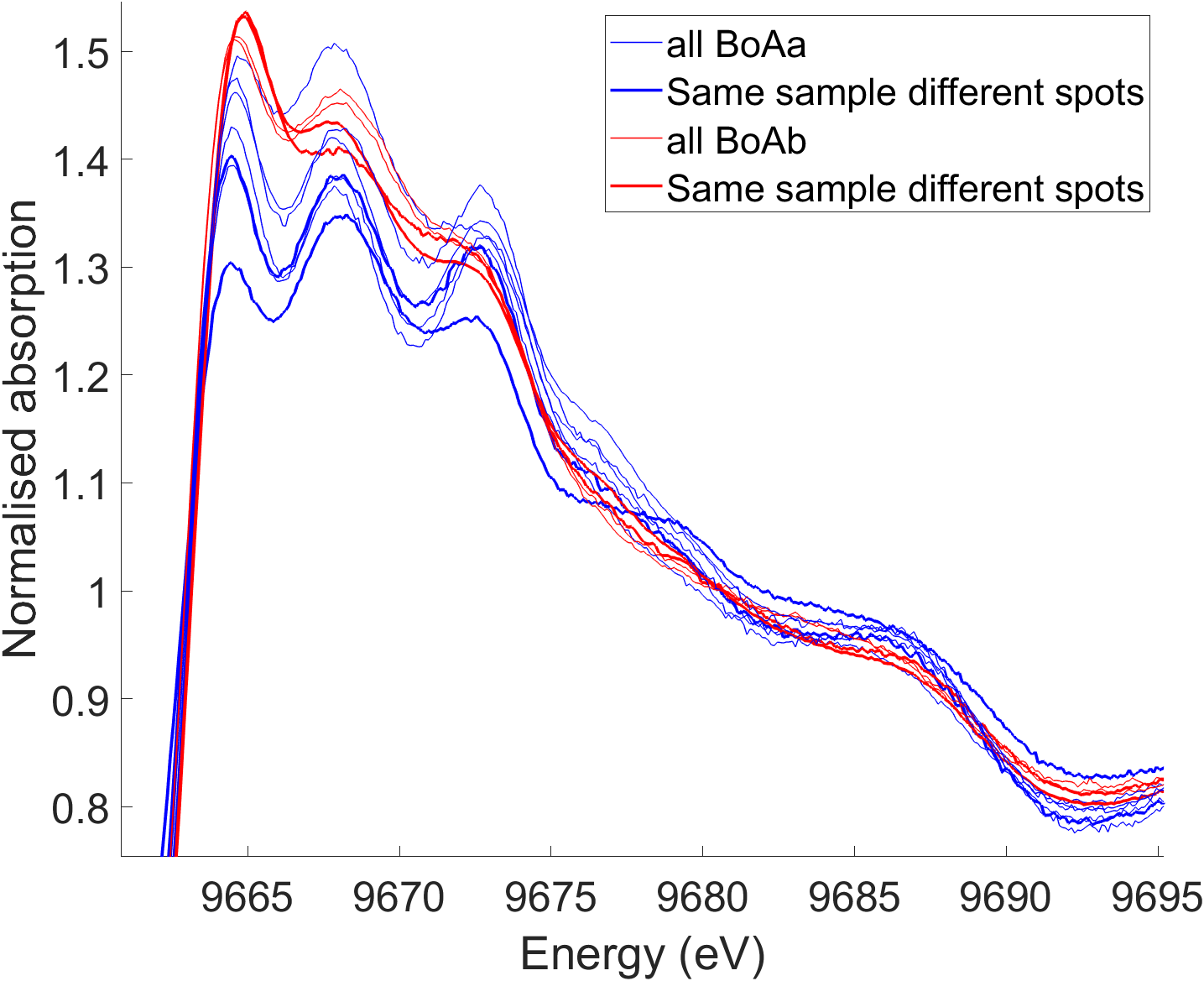


**Figure S3:** Boiler ash, BoAa and BoAb. Bold lines show spectra from the same sample but measured at different sample spots.

**Table S4:** LCF results for fine mode samples measured at different time points.

| Sample | K_2_ZnCl_4_ | KZnCl_3_·nH_2_O | Zn_5_(OH)_8_Cl_2_·H_2_O | R factor |
| --- | --- | --- | --- | --- |
| Fine1 (2 weeks after collection) | 0.99 | 8.4*10^-8^ | 0.012 | 0.00102 |
| Fine2 (6 months after collection) | 0.25 | 0.76 | 6.5*10^-9^ | 0.00063 |
| Fine3 (6 months after collection) | 0.11 | 0.89 | 5.1*10^-8^ | 0.00048 |
| Fine4 (1 year after collection) | 0.79 | 0.04 | 0.17 | 0.00357 |
| Fine1(1 year after collection, exposed to humidity) | 0.63 | 0.32 | 0.05 | 0.00226 |
| Fine2 (1 year after collection, exposed to humidity) | 0.98 | 3.9*10^-8^ | 0.02 | 0.00132 |

**The compounds used for the linear combination fits shown in figures 4 and 7:**

Coarse1: Zn_x+y_Ca_2-x_Al_2-y_SiO_7_ amorphous, Zn_Fhy, ZnAl_2_O_4_, ZnFe_2_O_4_, ZnCl_2_

Coarse2: Zn_x+y_Ca_2-x_Al_2-y_SiO_7_amorphous, Zn_Fhy, ZnAl_2_O_4_, ZnFe_2_O_4,_ NaZn_4_(SO_4_)(OH)_6_Cl·6H_2_O)

Coarse3: Zn_x+y_Ca_2-x_Al_2-y_SiO_7_ amorphous, Zn_Fhy, ZnAl_2_O_4_, ZnCl_2,_ ZnSO_4_·2H_2_O

Average BoAa: Zn_x+y_Ca_2-x_Al_2-y_SiO_7_ amorphous and crystalline, Zn_Fhy, ZnAl_2_O_4_, KZnCl_3_·nH_2_O

Average BoAb: Zn_x_glass, Zn_Fhy, ZnAl_2_O_4_, ZnFe_2_O_4_, ZnCl_2_

**References**

Leal, D. A., Machado Silva, G., Tedim, J., Wypych, F., & Marino, C. E. B. (2020). Synthesis and characterization of gordaite, osakaite and simonkolleite by different methods: Comparison, phase interconversion, and potential corrosion protection applications. *Journal of Solid State Chemistry*, *291*, 121595. <https://doi.org/https://doi.org/10.1016/j.jssc.2020.121595>

Machado Silva, G., & Wypych, F. (2022). A novel and facile synthesis route for obtaining highly crystalline impurity free layered hydroxide sulfates: Gordaite and osakaite. *Inorganic Chemistry Communications*, *143*, 109723. <https://doi.org/https://doi.org/10.1016/j.inoche.2022.109723>

Majerová, M., Škrátek, M., Prnová, A., Dvurečenskij, A., Kraxner, J., Švančárek, P., Cigáň, A., Maňka, J., & Galusek, D. (2019, 27–29 May 2019). Preparation and Characterization of Ni Doped Ca2Al2SiO7 Glass Microspheres. 2019 12th International Conference on Measurement,

Maruyama, S. A., Krause, F., Filho, S. R. T., Leitão, A. A., & Wypych, F. (2017). Synthesis, cation exchange and dehydration/rehydration of sodium gordaite: NaZn4(OH)6(SO4)Cl·6H2O. *Applied Clay Science*, *146*, 100–105. <https://doi.org/https://doi.org/10.1016/j.clay.2017.05.029>

Moezzi, A., Cortie, M. B., & McDonagh, A. M. (2013). Zinc hydroxide sulphate and its transformation to crystalline zinc oxide [Article]. *Dalton Transactions*, *42*(40), 14432–14437. <https://doi.org/10.1039/c3dt51638e>
